# A review of swarm-based metaheuristic optimization techniques and their application to doubly fed induction generator^☆^

**DOI:** 10.1016/j.heliyon.2022.e10956

**Published:** 2022-10-07

**Authors:** Kumeshan Reddy, Akshay K. Saha

**Affiliations:** Discipline of Electrical, Electronics & Computer Engineering, University of KwaZulu-Natal, 238 Mazisi Kunene Road, Durban, 4041, South Africa

**Keywords:** Current control, Torque control, Doubly fed induction generator, Optimization methods, Algorithms

## Abstract

In this paper, a review of Metaheuristic Optimization Techniques (MOT) which are currently in use for optimization in a vast range of problems, is presented. MOT are known for their simplicity and stochastic nature and successfully applied to solve complex engineering problems. Although there exist various categories of MOT, the techniques from swarm intelligence is reviewed in this paper. An explanation of the theoretical foundation upon which each algorithm is based is provided, along with the relevant mathematical models that explain how an algorithm attempts to obtain the best solution to a problem. The paper also reviews the applications of swarm-based MOT to the control of the doubly fed induction generator (DFIG). Particular attention is given to control of the DFIG for wind energy applications. Control of the DFIG is generally realized via the use of PI controllers. While various PI controller tuning methods are well established (such as the Ziegler–Nichols and Cohen–Coon methods), these methods produce satisfactory results, and often fail to meet the stringent levels of control presently required. Due to this fact, as well as the current success of MOT in engineering, the application of MOT to the control of the DFIG could be promising area of research. The results of the study show that although the various swarm-based MOT differ from each other in terms of aspects such as complexity and advantages, they are all based on the concept of randomness, and always attempt to produce the best possible solution. It was also observed that various swarm-based MOT displays the demerit of getting easily trapped in the local optimum, however various advancements have been proposed to correct such an issue. Based on the results of the application of these techniques to other engineering problems, their application to the DFIG could yield exceptional results.

## 1 Introduction

### 1.1 Motivation and incitement

**Figure 1:**
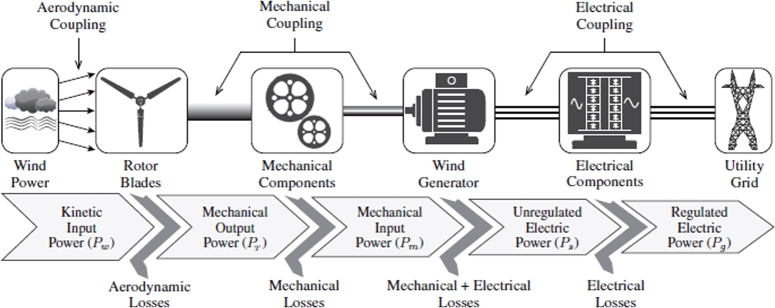
Conventional topology of WECS [3].

The world is currently in energy despair. For decades, the production of electrical energy depended on fossil fuels, particularly coal. An abundance of coal meant no limit of the utilization of this fuel to produce electricity. However, in recent times, this fuel source has come under investigation. This occurred for two reasons. The first reason is the scarcity of such fuel. The construction of many industries, urbanization and increasing population has led for a greater demand of electricity. This, in turn, has caused coal to be depleted at an alarming rate Secondly, due to the intense use of coal for the purpose of electricity production, the harmful effects of this fuel on the atmosphere have become more pronounced [1]. These issued have paved the way for the introduction of renewable energy. Due to its cost effectiveness, wind energy conversion systems (WECS) are gaining widespread attention. WECS utilize both asynchronous and synchronous machines [2]. The principle of producing electricity from WECS remains the same as that of a conventional power plant, the difference being the source of mechanical power utilized in driving the prime mover. In WECS, the wind turbine blades are attached to the rotor of the generator. This allows the rotation of the wind turbine blades to be transferred to the rotor of the machine. Generally, there is an interface, usually in the form of a three-stage gearbox, between the blades and the rotor. This allowed the low rotational velocity of the blades to be converted to a higher and more usable velocity. With the addition of various control actions, this rotational speed is converted into electricity. Evidently, WECS comprises of various mechanical and electrical components. The relationship of such can be observed in Fig. 1 [3].

Due to their robust nature, cheap maintenance costs, and large power generation capabilities, WECS are rapidly becoming the alternative to fossil fuel-based power generation. Moreover, the use of direct grid connected generators ensures that the aspect of grid inertia is present, which is a critical part of power system stability. It can then be observed that research in the control of WECS is of utmost importance, and should be carried out extensively.

### 1.2 Literature review

**Figure 2:**
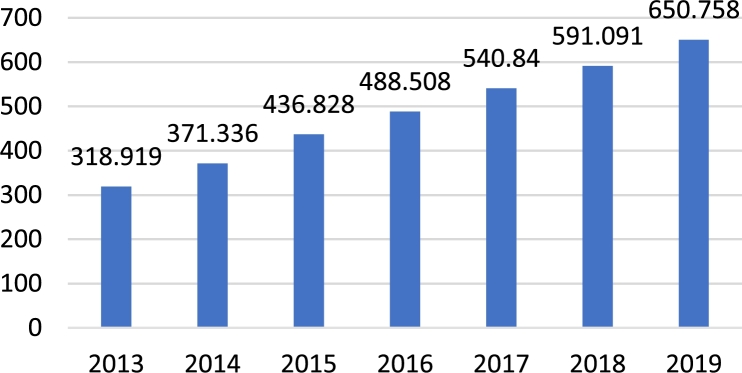
Total global capacity of wind energy systems (in GW) from 2013–2019 [5].

**Figure 3:**
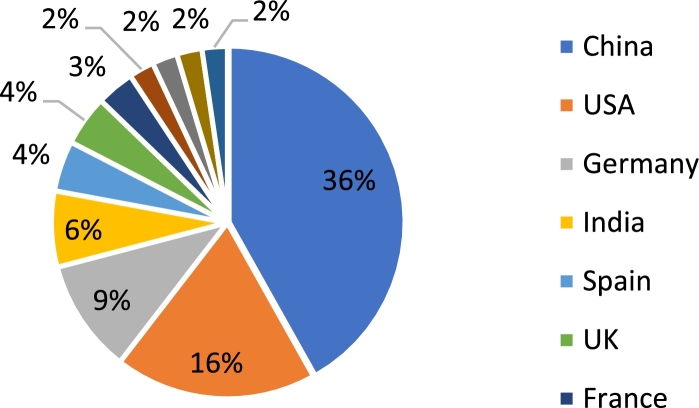
Participation of wind energy to global capacity of various nations [6].

**Figure 4:**
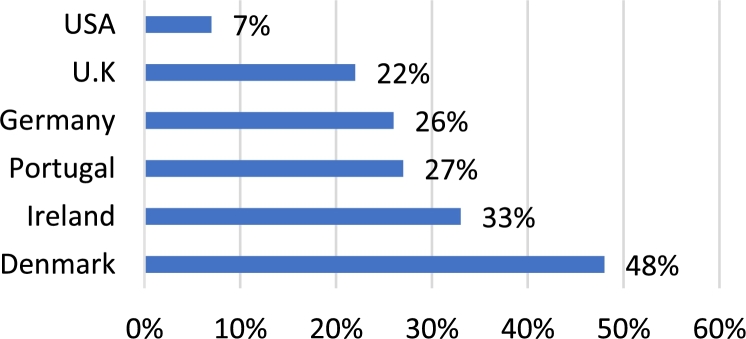
Infiltration of wind energy systems of several countries (%) [7].

The total global installed capacity of WECS has rapidly increased in modern times [4]. From 2013 to 2016, there has been a continual expansion in such systems. This is observed in Fig. 2 [5]. The two leaders in implementation of such systems are the United States of America and China. With an installed capability of 237 MW, China is ahead by a significant margin. The United States has a capacity of approximately 106 MW, with Germany coming in third with a capacity of 62 MW [6]. Further, it should be noted that various European and Asian nations have recently exhibited a rapid increase in the installation of such systems [6]. This can be seen in Fig. 3, which depicts the magnitude of wind energy contribution from the top ten countries [6]. However, despite China and the USA yielded a larger magnitude of installed capacity, the total contribution of such systems to the total national energy consumption is only a fraction. This is observed in Fig. 4 [7]. Also from Fig. 4, it can be seen that despite European nations yielded a smaller magnitude of energy, this energy accounts for a much larger percentage of the total national energy consumption [5], [8]. The important fact, though, is that there has been a sharp rise in the utilization of wind energy for the production of electricity. This points to a green and sustainable future. In wind farms, the generator most commonly utilized are the DFIG and permanent magnet synchronous generator [9]. This is due to their capability to produce the maximum possible power, despite fluctuations in the wind velocity. Also, when compared to the squirrel cage induction machine, these machines produce a lower level of stress on the machine components.

Despite the advantages of the PMSG, the DFIG proves to be the more efficient generator. Thus, majority of WECS utilize the DFIG [10], [11]. However, recent research has been conducted in the control of the PMSG. One such example can be found in [12], whereby a novel MOT, called Democratic Joint Operations Algorithm, was utilized for the purpose of obtaining PID controller gains. When compared to various other algorithms, the proposed algorithm produced the best overshoot and steady state error of the active power. The authors in [13] propose an adaptive Fractional Order PID controller for Maximum Power Point Tracking (MPPT) which utilizes a linear perturbation observer. The controller is easy to implement, does not require an accurate model, and exhibits a robust control performance. Owing to the rapid increase in the use of the DFIG, control of such needs to be efficient and effective. The most common and established DFIG control method is field oriented control. This control algorithm regulates the DFIG stator active and reactive power via control of the rotor current [14], [15]. This utilizes proportional-integral (PI) controllers. PI controllers are known to produce reliable and robust responses. The issue, however, is that PI controllers are required optimal tuning.

Achieving this via trial and error is a tedious task and may result in sub-optimal performance of the controller. One well-known method of PI controller tuning is the Ziegler–Nichols method, which utilizes either the closed-loop or open-loop response of the plant. Another method is the Cohen–Coon tuning method, which is similar to that of the Ziegler–Nichols method but makes use of different formulae to determine optimal controller performance [16], [17], [18]. These methods prove to be satisfactory, but often cease to meet the stringent levels of control presently required. This becomes an issue with grid code compliance, especially under abnormal conditions such as symmetrical and asymmetrical grid voltages, and fault ride through.

Recently, several alternatives to the PI controller have surfaced. One such method is Sliding Mode Control (SMC). In SMC, a pre-set trajectory is utilized along which the control variable is forced [3]. SMC offers robustness to parameter variations, external disturbances, nonlinear loads and uncertainties [19], [20]. However, it suffers the demerit of chattering [21], [22]. Hysteresis control makes use of user defined bandwidths. The output of the hysteresis controllers is used to determine which converter switching state will be implemented [23]. This is commonly referred to as a Look-Up Table. This is a simple control method. When applied to the DFIG, it proved to provide efficient dynamic responses. However, the ripple in the output is extremely large, and the output of the stator current is severely distorted [24]. Artificial Neural Network (ANN) is a system which is based on the human central nervous system. ANN simulates a biological neural network [25], [26]. The merit of ANN includes the ability to work with incomplete knowledge and having a strong fault tolerance. However, ANN suffers the demerit of an unexplained behavior of the network. This compromises the reliability of the network [27]. Further, ANN is known to have a greater than average computational burden [27], [28]. Model Predictive Control has been extensively applied in the process control industry and has recently shown promise in the field of electrical engineering [29]. It offers a simple structure but is built on the knowledge of accurate machine parameters [3]. Practically, machine resistance and inductance values are given in terms of a tolerance, making Model Predictive Control an unreliable control method. When applied to the DFIG, it is observed that MPC produces a large steady state error, particularly at lower shaft angular velocities [30].

### 1.3 Contribution and paper organization

Considering the control of a DFIG, aspects such as frequency and reactive power absorption/generation are required to have an extremely low error tolerance. This is important for the efficient operation and stability of the electrical grid. Poor control of these critical aspects may have catastrophic consequences. In addition to this, national grid code requirements are required to be met. The aim of this research is to thoroughly investigate the effect of utilizing MOT in the control of the DFIG. In this paper, swarm-based MOT are considered, with fourteen techniques investigated. Each technique is investigated in terms of inception, mathematical modelling, application procedure, merits, demerits and advancements of such and finally the application of these techniques to the control of the DFIG. This paper provides a general review of the techniques and is aimed at researchers interested in the control of DFIG based WECS. After the structure of each algorithm, the merits, demerits and advancements of each algorithm are presented. This has an indirect correlation with the application of these techniques to the control of the DFIG. The presented literature concerning algorithm advancements, in combination with the presented literature regarding the application of swarm-based MOT to DFIG control, will equip the researchers with sufficient knowledge to utilize a specific technique advancement in the application of DFIG control. This paper, therefore, serves as a basis for scientific advancement concerning DFIG based WECS control.

**Figure 5:**
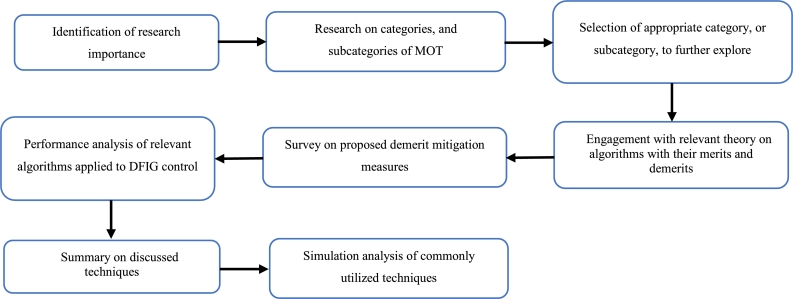
Methodology process for review conducted.

Other articles which focus on the review of swarm-based MOT exist but discuss only a few algorithms. Further, there is no comprehensive review on the effect of the advancements of the conventional algorithms. Lastly, a review on the use of swarm-based MOT in regard to application to DFIG control has not been reported where the contribution of this paper is focused. Advancements to conventional algorithms, as well as the application of the algorithms to DFIG control, are critically reviewed and analyzed. The balance of this article has the following structure. Following the introduction, an overview of the DFIG is given. This is in terms of structure, modelling and control methods. Thereafter, an overview of MOT is given. This is in terms of classification and application to the control of the DFIG. Afterwards, the fourteen different swarm-based MOT are investigated in-depth. This is in terms of mathematical modelling, method of application, merits, demerits and advancements to overcome such, and finally the application of these techniques to the control of the DFIG. Following this, a table that summarizes all of the information captured in chapter 4 is presented. In section 6, a simulation-based performance analysis of common algorithms is presented followed by a conclusion and scope of future work in section 7. Fig. 5 depicts the method followed in order to realize this review study. The main contributions of this paper are as follows:

• Provide a comprehensive review of the principle of operation of fourteen swarm-based MOT. This includes the provision of relevant equations required for execution of the algorithm, as well as a flow chart depicting the steps to successful application of the algorithm.
• Provide a review on the merits and demerits of each algorithm, as well as a review on recent advancements for mitigation of relevant demerits.
• Analyze the application of such algorithms for the sake of DFIG control. This includes an identification of gaps in current literature.
• Carry out a simulation-based performance analysis of three popular swarm intelligence techniques. This analysis is in terms of exploration capability, exploitation capability, and convergence rate.

## 2 An overview of the DFIG

**Figure 6:**
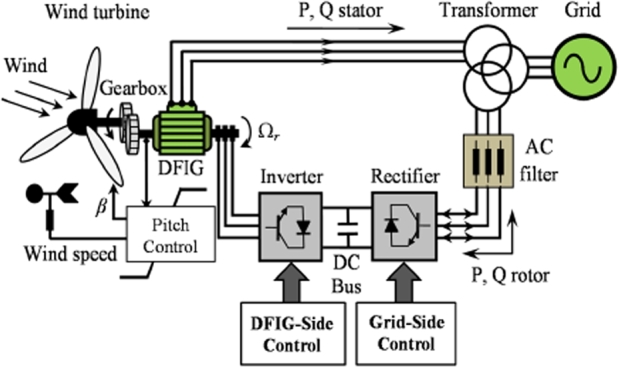
DFIG based WECS [3].

The structure of the DFIG based WECS is such that the stator provides a direct grid connection, and the rotor makes use of a back to back converter to provide grid coupling. This unique rotor configuration allows the rotor to both absorb and supply electrical power, thus allowed for generator operation at any wind speed [31], [32]. The rotor supplies power at speeds greater than synchronous speed and absorbs power at speeds lower than synchronous speeds [32]. To ensure a constant output frequency, power is absorbed at slip frequency [33]. Fig. 6 depicts the structure of the DFIG-WECS [3].

**Figure 7:**
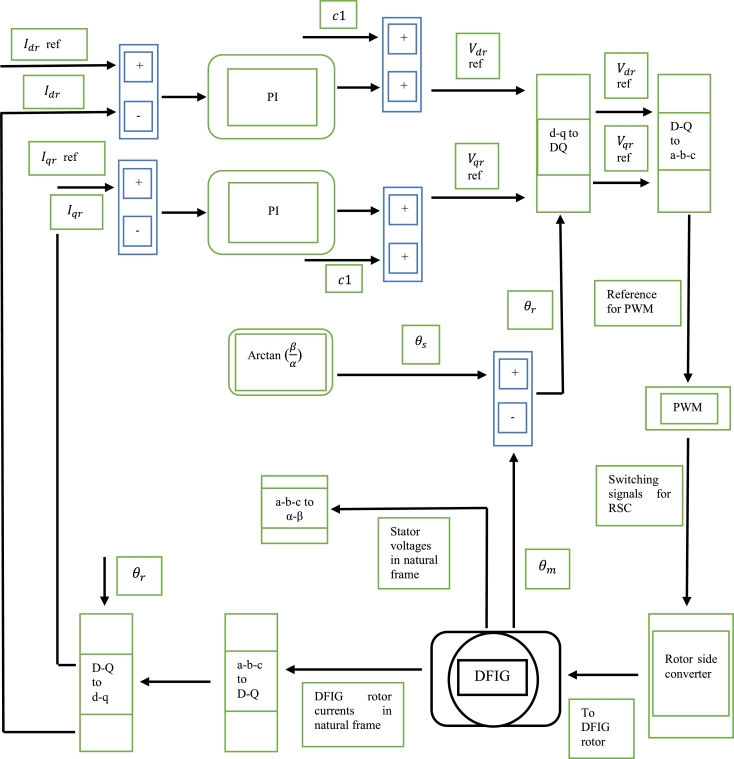
Structure of FOC of DFIG [39].

Considering the synchronous (d-q) reference frame, the DFIG voltage equations can be seen in [34], [35]. The DFIG rotor and stator fluxes can be represented as shown in [36]. The DFIG electromagnetic torque, as well as the stator and rotor active and reactive powers are shown in [37] and [38] respectively. When considering DFIG control, various control strategies exist. These are rotor current control, direct power control (DPC) and direct torque control (DTC) [34]. Rotor current control is conventionally achieved via field-oriented control (FOC). FOC utilizes PI controllers to regulate the rotor currents, thus allowing for indirect control of the stator active and reactive powers [35]. This is the most common method of control. The function block diagram of stator voltage FOC is observed in Fig. 7. In Fig. 7, c1 and c2 denote the coupling terms of the algorithm. The algorithm is explained in detail in [39]. DTC directly controls the generator rotor flux and torque. There are two ways in which conventional DTC is achieved. In method one a look up table and hysteresis controllers (DTC-ST) are utilized. The second way involves the use of PI controllers [40], [41]. DPC control directly the stator reactive and active powers. DPC is achieved using the same methodology as in DTC-ST [42].

## 3 Metaheuristic optimization techniques

**Figure 8:**
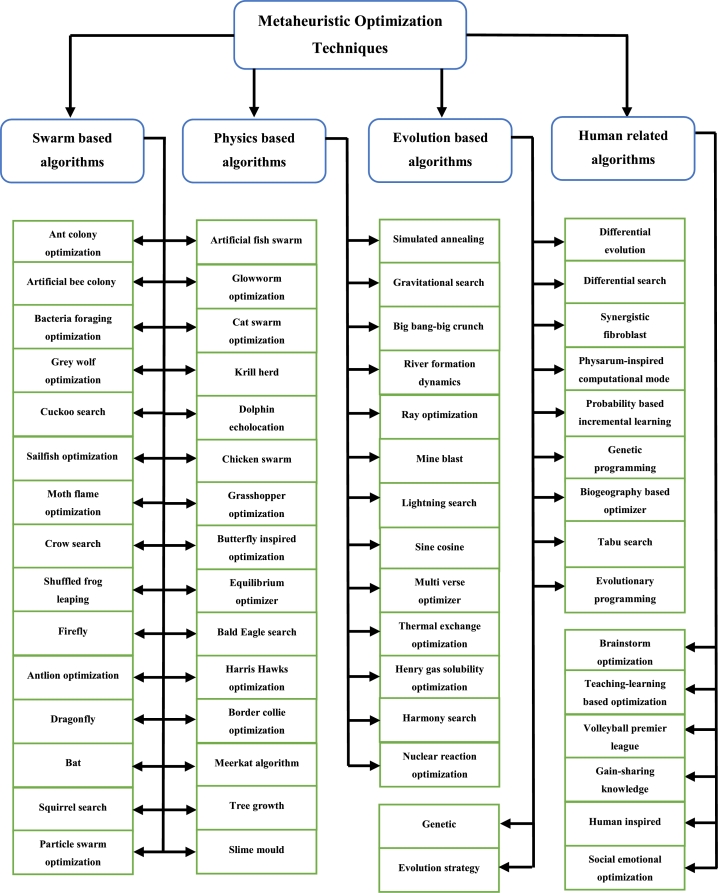
Flow chart indicating classification of MOT [44], [45].

Metaheuristic Optimization Techniques, as the name suggests, are problem independent control techniques which has gain rapid popularity in the application of complex engineering problems. This can be attributed to their simplicity, flexibility, and capability to solve complex problems at a high efficiency rate. Metaheuristics techniques are based strongly on the concept of randomness, and search for optimal solutions based on diversification and intensification. Diversification is the scattered search of an entire search space and intensification is the search in a particular area of a search space [43]. MOT are based on various aspects of everyday life, such as the human body, the laws of physics and the behavior of animals in their natural habitat [44]. Critical evaluation of the working processes of these aspects has allowed for accurate mathematical modelling of various nature-based occurrences. This in turn has been used to solve complex engineering problems successfully and optimally. While there does not exist any definitive way to categorize MOT, it can usually be classified into four categories [44], [45]. This can be seen in Fig. 8. The classification shown in Fig. 7 is not an exhaustive list of MOT but does account for most of the currently implemented techniques. The application of MOT has recently been applied to the control of the DFIG but has not been extensively researched. It has mostly been used to optimize the controller gains of the PI controllers used in the control of the DFIG. It is shown later that MOT make use of fitness functions. In terms of proportional-integral (PI) controllers, the various fitness functions (performance indices) are time varying functions of the integral of either the square of absolute value of the error being input into the PI controller [46], [47], [48].

Metaheuristic optimization techniques have been applied extensively to the field of renewable energy systems. In [49], a critical survey on the application of Metaheuristic optimization techniques on proton exchange fuel cell parameter estimation was carried out. The survey considered MOT from all four categories, both in conventional and modified natures, thereby making the survey extensive. Considering application to photovoltaic (PV) systems, the authors in [50] carried out a survey that reports the effects of utilizing MOT for identification of PV cell parameters. As in [49], the paper considered techniques from all four categories, both in conventional and modified natures. The paper outlined in [51] conducts a comprehensive survey on the effect of MPPT algorithms of PV systems under the effect of partial shading. Seven categories of algorithms are considered, one of which is MOT. Within this, three of the four subcategories of MOT are discussed. Further to discussion of the conventional algorithms, the paper acknowledges the utilization of hybrid algorithms for the said application.

## 4 A review of various swarm-based MOT

The following section provides a review on various swarm-based MOT. The techniques that will be considered are Particle Swarm Optimization, Bacteria Foraging Optimization, Grey Wolf Optimization, Artificial Bee Colony Optimization, Whale Optimization Algorithm, Crow Search Algorithm, Bat Algorithm, Squirrel Search Algorithm, Moth Flame Optimization, Sailfish Optimization, Cuckoo Search Algorithm, Firefly Algorithm, Shuffled Frog Leaping Algorithm and Antlion Optimization. Each technique is discussed in terms of motivation, structure, merits, demerits, advancements and finally their application to the control of the DFIG.

### 4.1 Particle swarm optimization

Utilizing the social conduct of the schooling of fish and the flocking of birds as inspiration, particle swarm optimization (PSO) is a MOT which, in was developed by an electrical engineer and social psychologist. PSO consists of a population of particles which move at a given velocity. The velocity of every particle is updated after each iteration. These updates consider various factors. The aim of the motion of the particles in the population is the move to the most ideal solution of the problem. PSO is a simple control algorithm which has a light computational burden [52], [53], [54], [55]. Considering the real number space, a particle can be defined as a possible solution which moves through the search space of the problem. The position of a particle is a function of the particles previous position and current velocity.

This can be expressed as [52], [56], [57]:

(1) $${\bar{x}}_{i}(t+1)={\bar{x}}_{i}(t)+{\bar{v}}_{i}(t)$$

Where ${\bar{x}}_{i}(t+1)$ is the updated position of the particle, ${\bar{x}}_{i}(t)$ is the current position of the particle and ${\bar{v}}_{i}(t)$ is the current velocity of the particle.

The current velocity of the system is defined as [52], [56], [57]:

(2) $${\bar{v}}_{i}(t+1)={\bar{v}}_{i}(t)+\sigma_{1}\times rand1\times ({\bar{p}}_{i}-{\bar{x}}_{i}(t))+\sigma_{2}\times rand2\times ({\bar{p}}_{g}-{\bar{x}}_{i}(t))$$

Where ${\bar{v}}_{i}(t+1)$ is the updated velocity of the particle, $\sigma_{1}$ and $\sigma_{2}$ are two positive numbers, rand1 and rand2 are two randomized numbers in the range [0,1], ${\bar{p}}_{i}$ is the individual best of each particle and ${\bar{p}}_{g}$ is the global best of each particle. As shown, (2) comprises of three elements. The first term is based on the inertia of the particle (according to newtons first law, a body in motion tends to continue motion unless disturbed by an external force) [28]. The second term describes the particles propensity to gravitate towards its personal best. It is known as the memory component. The third term describes the particles propensity to gravitate towards to global best i.e., the best of all the particles. It is known as the social component [52], [57]. The individual best and global best are obtained based on a fitness function which is defined by the user [52]. To ensure convergence of the particles and prevent divergence (going to infinity), selection of appropriate constants and setting limitations is essential. One of the critical limitations that needs to be present in the selection of a maximum velocity. A too large maximum velocity could result in unstable behavior of the particles and a too small velocity limits the search space and could result in the most optimal solution not being discovered. An experiment performed in [58] proved that by dynamically changing the maximum velocity, the performance of the algorithm can be enhanced. Another important limitation is the values of the acceleration constants, $\sigma_{1}$ and $\sigma_{2}$. In a study conducted in [59] and [60], it is shown that if the sum of $\sigma_{1}$ and $\sigma_{2}$ exceed 4, the particle trajectory diverges (goes to infinity). The values of the acceleration constants can be updated dynamically, in which case they are calculated based on a maximum and minimum value, as well as the current and maximum iteration numbers [61].

However, even if the acceleration constants and maximum velocity are selected correctly, there is a possibility that the particles would continue to diverge. To prevent this, there exists two methods which can be applied to (2). The first method is applying a constant called the constriction factor. This is applied to the entire of (2) and is based on the use of the two acceleration constants [56]. The second method is accomplished by applying either a fixed or dynamic value only to the inertia component of (2) [56]. This is termed inertia constant and usually begins at a high value and gradually decreases. Considering a dynamic inertia constant, *w*, the constant is calculated using an initial weight (usually 0.9), a final weight (usually 0.4), as well as the current and maximum iteration numbers [47], [56], [62]. The suitable selection of the inertia weights results in the requirement of a smaller number of iterations to obtain an acceptable solution [62].

**Figure 9:**
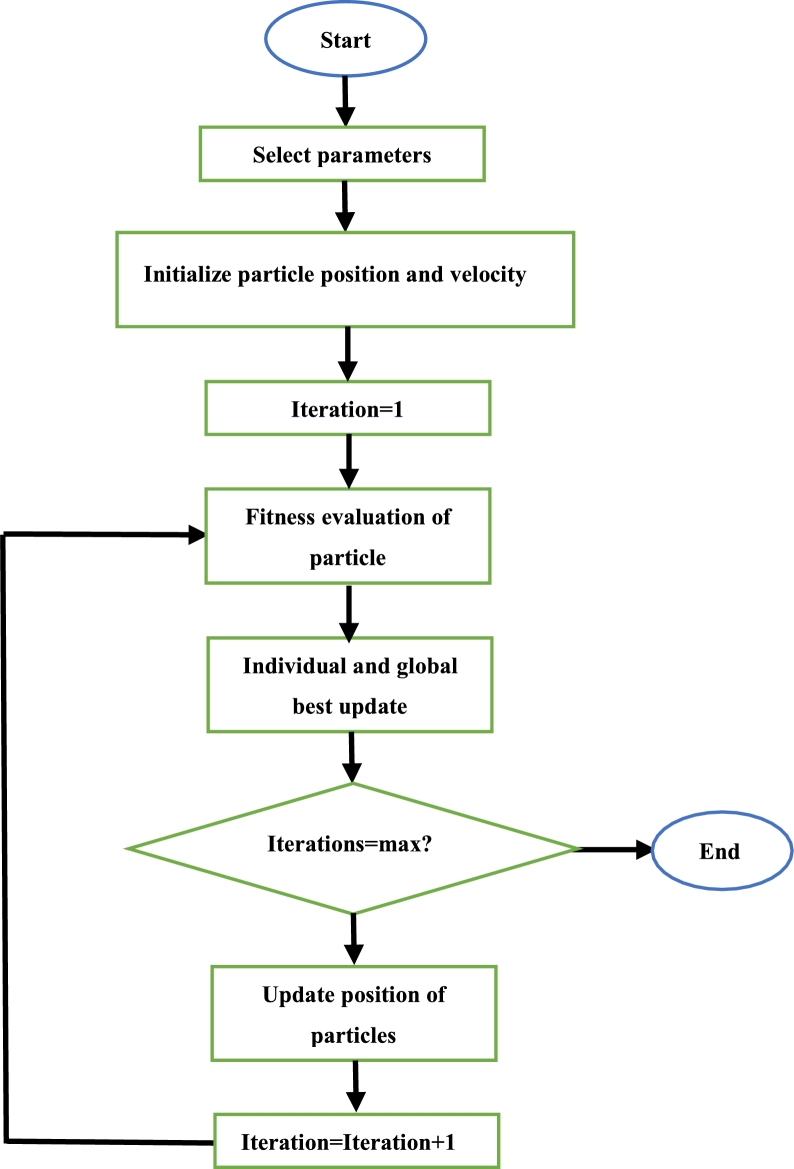
PSO algorithm flowchart [63].

At first, the relevant parameters (number of particles, iteration number, initial acceleration constant and initial inertial weight) are defined. Then, each particle positioned randomly throughout the search space. The next step is the evaluation of the fitness of each particle. The particle which has the lowest fitness function is determined and the position of that particle is taken as the global best. Thereafter, the position of each particle is then updated using (2) and the fitness function is evaluated again. This fitness function value of each particle is then compared to the previous fitness function of that particle. If the current fitness function is superior to the previous fitness function, this value replaces the old value, and the current position replaces the previous position as the new individual best. This process continues until the iterations are complete. Once this is so, the particle with the best fitness function is said to be the best solution [52], [57]. The steps to execute the PSO algorithm is depicted in Fig. 9 [63].

PSO was initially designed to solve continuous nonlinear functions. However, in cases where binary and integer values must be arranged as particles, the PSO algorithm can be adapted to perform this [52], [55]. In binary particle swarm optimization (BPSO), the same equation as shown in (2) is used. From this calculation of velocity, a probability is determined and is defined as [64]:

(3) $$S(v_{i}(t+1))=1/(1+\exp(-v_{i}(t+1)))$$

A randomized number $x_{r}$ in the range [0,1] is generated. If the probability defined in (3) is greater than the randomized number, the particle takes on a position of 1. If not, the particle takes on a position of 0 [64], [65]. The personal and global best can be updated as follows [65]:

(4) $${\bar{p}}_{i}(t+1)=\left\{ \begin{array}{cc} x_{i}(t+1),\; & \text{if }F(x_{i}(t+1))<F({\bar{p}}_{i})\\ {\bar{p}}_{i}(t),\; & \text{otherwise} \end{array}\right.$$

(5) $${\bar{p}}_{g}(t+1)=\left\{ \begin{array}{cc} p_{i}(t+1),\; & \text{if }F(p_{i}(t+1))<F({\bar{p}}_{g})\\ {\bar{p}}_{g}(t),\; & \text{otherwise} \end{array}\right.$$

Where *F* is the fitness function. Despite its merit of a fast convergence speed [66], [67], the conventional PSO suffers the demerits of poor accuracy and being easily trapped in the local minima [66], [67], [68]. The authors in [66] introduced a mutation factor and a dynamic inertial factor. Large inertial factors enhance the convergence rate of the algorithm while small inertial factors enhance the search accuracy. The proposed dynamic inertial factor is a function of the fitness of all the particles, and lies in the range [0,1]. Based on the position of all particles except the global best, the mutation factor randomly generates two new particles based on a probability. The proposed algorithm was applied to the 14-BUS system for reactive power optimization and compared to the conventional PSO. It was observed that after 100 iterations, the proposed algorithm produced a 1.28% improvement in the result. For iterations lower than four, MPSO is inferior to PSO. However, in practice, such minute values of iterations are not utilized.

Considering the application of PSO to the DFIG, the authors in [61] applied PSO to the optimization of the parameters in sliding mode control (SMC). Three different control algorithms were presented. The first algorithm was the conventional SMC, in which PSO was used to optimize the positive switching gain. The second algorithm was the integral SMC, which is an advancement to the conventional SMC in the case of reduction in steady state error. In this algorithm, PSO was used to optimize both the positive switching gain and integral gain. The third algorithm was an intelligent sliding mode controller, which adds a proportional gain to the integral sliding mode controller. In this algorithm, PSO was used to optimize the positive switching gain, integral gain, and proportional gain. The proposed control methods were implemented using the rotor current control method, which means that the control of the DFIG stator active and reactive power was dependent on the control of the rotor direct and quadrature currents. The proposed control methods were tested on a 7.5 kW DFIG. From the results, it is observed that the integral sliding mode controller produced the best dynamic response for both the stator reactive power and active power. This is followed by the intelligent SMC, then the conventional SMC. The superiority was in the order of approximately 100% and 200% to the intelligent SMC and conventional SMC respectively. The results presented do not clearly show a difference in steady state error and steady state ripple among the three control algorithms. Furthermore, the results are not compared to other parameter optimization methods, such as other MOT or the Ziegler Nichols method. This means that the results of the proposed algorithms cannot be verified.

PSO is applied to the DFIG to analyze the small signal stability in [69]. PSO is used to optimize the PI controller gains for both the rotor side converter (RSC) and grid side converter (GSC), and the pitch controller. In total, twelve parameters were optimized. The system was tested on both small and large disturbances. For the small disturbances, the optimized controllers produced smaller overshoots for the dc link voltage, terminal voltage, stator reactive power and stator active power. The optimized controller also damped out the oscillations much quicker. The author claims that the optimized controllers produce a superior dynamic response but due to insufficient evidence, this claim cannot be validated. Considering large disturbances, the optimized controllers produced a better terminal voltage and lower peak dc link voltage. With regards to the stator active power, the optimized controllers continued to inject active power into the grid, whereas the un-optimized controllers failed to produce any active power. Considering the stator reactive power, the optimized controllers absorbed a lower amount of power.

An advancement of [40] was carried out in [70], where sensitivity analysis is utilized to identify the unified dominant control parameters. These are the parameters that would be optimized using PSO, so that the algorithm intricacy is lessened. The authors make use of trajectory sensitivity, which measures the degree of change of a system based on a differential change on a specific parameter and eigenvalue sensitivity, which uses eigenvalues to determine the systems sensitivity towards a specific parameter. Using the trajectory sensitivity analysis, the integral gain of the grid voltage regulator and the proportional gains of both the direct and quadrature rotor current regulators were chosen as the dominant control parameters. Using the eigenvalue sensitivity analysis, the integral gain of the stator active power regulator, integral gains of both the direct and quadrature rotor current regulators, proportional gain of the dc link capacitor voltage regulator and the proportional gains of both the direct and quadrature grid current regulators were chosen as the dominant control parameters. In total, there were six parameters to be optimized using PSO. The proposed algorithm (UDCP-PSO) was tested on both a single machine bus system as well as a multi machine bus system for both small and large disturbances. The proposed algorithm is fared against the original ten parameter optimization algorithm (C-PSO) as well as a random parameter optimization algorithm (R-PSO).

Considering the single machine bus system under a small disturbance, the UCDP-PSO produced a smaller overshoot of stator active power when compared to C-PSO and R-PSO. The damping time for UCDP-PSO was the same as C-PSO, which was superior to R-PSO. The dynamic response of all three is almost identical. For the stator reactive power, the percentage overshoot and damping time of UCDP-PSO and C-PSO are the same and superior to R-PSO. The dynamic response of all three is almost identical. An identical phenomenon was observed with respect to the dc link voltage. Considering the single machine bus system under large disturbance, the UCDP-PSO produced the smallest percentage overshoot. The damping time and dynamic response of all three algorithms appear to be the same, with any variance being negligible. An identical response was seen with regards to the stator reactive power, this time the UCDP-PSO algorithm producing superiority only marginally to C-PSO. Considering the dc link voltage, UCDP-PSO once again produced the smallest percentage overshoot. UCDP-PSO and C-PSO produce an identical damping time, which was superior to that of R-PSO. The dynamic response of all three algorithms was identical.

Considering the multi machine bus system under small disturbance, UCDP-PSO and C-PSO produce the same percentage overshoot and damping time, which was superior to that of R-PSO. The dynamic response of all three algorithms was the same. For the stator output voltage, C-PSO produced the lowest overall percentage overshoot, with the damping time of UCDP-PSO and C-PSO being the same and superior to that of R-PSO. Once again, the dynamic response of all three algorithms was the same. A conventional PID control of a DFIG using PSO is implemented in [71]. However, the fitness function used was not a conventional one (such as ITAE), but rather a unique one. This unique fitness function is a function of the steady state error, settling time, rise time and overshoot. The proposed control algorithm was compared to the supervisory PID control method. The DFIG terminal voltage is dropped from 1 per unit to 0.5 per unit, before regaining to 1 per unit. The results showed that the proposed algorithm outperformed the supervisory control method in all aspects i.e., settling time, rise time, peak time, and percentage overshoot. The unbalance in the stator currents during the voltage drop was approximately the same for both the proposed algorithm and the supervisory control method.

A novel control structure for stability enhancement of a DFIG based ocean energy conversion system is proposed in [72]. The structure of the control lies in the basis of a Function Link-based Wilcoxon radial basis function network (FLWRBFN). The learning rates of FLWRBFN were tuned using a hybrid Differential Evolution and PSO technique. The study aimed at analyzing the dynamic and transient performance of wave power generation systems under disturbances and grid fault. The proposed algorithm was compared to the PI controller and radial basis function network (RBFN). Considering the turbine speed, line voltage, dc link voltage and grid side real power, the FLWRBFN achieved a lower overshoot and faster settling time for all aspects for both the dynamic and transient responses. It was also observed that the FLWRBFN with PSO-DE also produced the overall best convergence rate. PSO was applied to a DFIG based dish Stirling system in for maximum power point tracking and regulation of receiver temperature in [73]. A control scheme based on average pressure control and coordinated torque was proposed. This proposed model required only four parameters to be optimized, compared to twenty in existing control schemes. These four parameters were optimized using PSO. The results showed that as irradiance varied, the proposed control scheme was superior in providing reactive power to the grid as well as achieving temperature regulation.

### 4.2 Bacteria foraging optimization

Bacteria foraging optimization (BFO) is a MOT inspired by the conduct of the E. Coli bacterium which is present in the human intestine. The motion of the bacterium is dependent on the motion of the flagella which is attached to the bacterium (as a tail). The bacterium either tumbles (changes direction with minimal displacement) or swims; if the flagella rotates clockwise then the bacterium tumbles and if the flagella rotate anticlockwise then the bacterium swims. There are four steps involved in BFO. These are chemotaxis, swarming, reproduction, and elimination-dispersal [74], [75], [76], [77]. This alternating motion of tumbling and swimming is known as chemotaxis. The aim of chemotaxis is to allow the bacteria to move towards nutrient rich environments and avoid noxious environments. The bacteria continue to swim in nutrient rich environments and tumble in noxious environments. Consider that $\theta^{i}(j,k,l)$ is a representation of the position of the $i^{th}$ bacterium, at the $j^{th}$ chemotactic, $k^{th}$ reproductive and $l^{th}$ elimination-dispersal step. Then [74]:

(6) $$\theta^{i}(j+1,k,l)=\theta^{i}(j,k,l)+C(i)\times (\frac{\Delta i}{\sqrt{\Delta^{T}(i)\Delta (i)}})$$

Where C(i) is the length of the taken step (which is in a random direction dependent on the tumble) and Δ*i* is a randomized vector in the range [−1,1]. Swarming is based on the observation of the behavior of various bacteria. It is based on their movement as a group towards nutrients. The bacteria either attract or repel each other, and this cell-to-cell signalling can be represented as [74], [76], [77]:

(7) $$J_{cc}(\theta ,P(j,k,l))=\sum_{i=1}^{S}J_{cc}(\theta ,\theta^{i}(j,k,l))=\sum_{i=1}^{S}[-D_{a}\exp(W_{a}\sum_{m=1}^{P}{\left(\theta_{m}-\theta_{m}^{i}\right)}^{2})]\;+H_{r}\exp(W_{r}\sum_{m=1}^{P}{\left(\theta_{m}-\theta_{m}^{i}\right)}^{2})]$$

Where $J_{cc}(\theta ,P(j,k,l))$ is objective function that is added to the original cost function so that the function becomes a function of time variance, *S* is the total number of bacteria present, *P* represents the number of variables to be optimized, $\theta ={\left[\theta_{1},\theta_{2}\ldots \theta_{p}\right]}^{T}$ represents a point which lies in the p-dimensional search domain, $D_{a}$ is the cell attractant depth, $W_{a}$ is the width of the attractant signal, $H_{r}$ is the magnitude of the repellent and $W_{r}$ is the width of the repellent signal. The aim of the algorithm is to obtain the least possible (minimal) cost function. In reproduction, the bacteria with the higher cost functions die off, and those with lower cost functions split into two and are situated in the same location. This ensures a constant population of bacterium, but of a better overall quality [74], [76]. Elimination and dispersal occur because of a nutrient rich environment suddenly becoming unfavorable to the bacteria. In this case, some bacteria die and some move to a new location. To simulate this behavior, some bacteria are killed off randomly and their replacements are placed in a random location [76]. A random number, *U*, in the range [0,1] is generated. Another random number, $P_{ed}$, in the same range is generated [74].

Replacements are positioned according to the following [74]:

(8) $$\text{If }U<P_{ed}\text{ then the replacements are randomly placed}\;\text{Else, the replacements are not placed}$$

**Figure 10:**
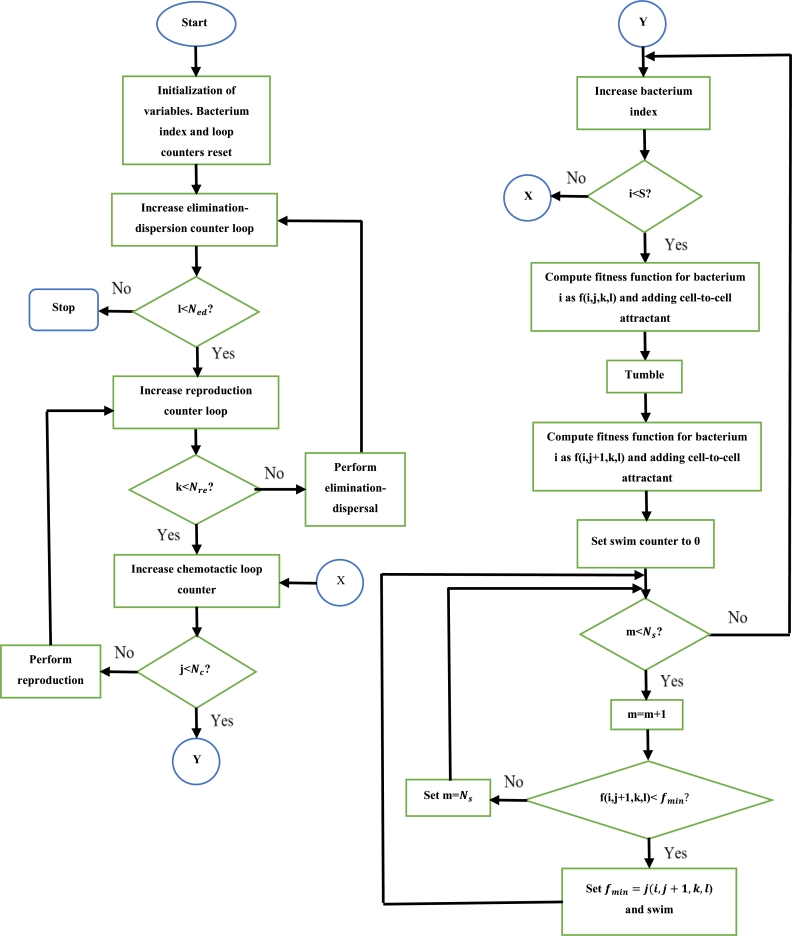
BFO algorithm flowchart [78].

Initially, the required parameters are defined. Then, each bacterium is randomly positioned. Thereafter, the fitness of each bacteria is computed. The position of each bacterium is then updated using (6), and the corresponding fitness is computed. If this fitness is superior to the previous fitness, then the position and fitness function is updated. If this is the case, then the bacteria's position is again updated, and the new fitness value is compared to the current fitness function value. This would occur until the maximum number of repetitions in a single chemotactic step is reached. This process is carried out for all the bacteria. Once this process has been completed for all the bacteria, the process is restarted, each time incrementing the number of chemotactic steps taken. This is continued until the maximum number of chemotactic steps are reached. Then, reproduction takes places where the unhealthy bacterium dies off and the healthy bacterium is split into two and occupies the same space. After this, the process is restarted by once again updating the position of the bacterium, this time incrementing the number of reproduction steps taken. This occurs until the maximum number of reproduction steps are taken. Then, some bacteria are randomly killed, and new bacteria are randomly placed in the search space (if the condition outlined in (8) is met). The process then starts from the beginning, by updating the positions of the bacterium. The number of elimination-dispersal steps taken is incremented. This entire process continues until the number of elimination-dispersal steps reaches its maximum value. At this point, the bacterium with the best fitness is chosen as the best solution [74]. The steps to execute the BFO algorithm can be seen in Fig. 10 [78].

One of the strong merits of the conventional BFO is that it does not easily get trapped in the local minima [79]. To the authors best knowledge, there has been no established demerits of BFO. This does not mean that none exist, but rather points to the lack of application of the algorithm. Considering the application of BFO to the DFIG, the authors in [80] make use of BFO to dampen low frequency oscillations. Both the GSC and RSC were considered. A PI based damping controller was added to the RSC. The control method considered the stator active power, stator voltage magnitude, dc link voltage and GSC reactive power consumption. The entire control algorithm made use of seven PI controllers and used BFO to optimize the gains of each controller. The objective function used was based on the damping ratio of the eigenvalues of the system, meaning that the control method makes use of the differential algebraic equations of the system. The PI controllers were optimized at three different wind speeds (7 m/s, 8 m/s and 8.5 m/s). For every wind speed, the DFIG is operated at the synchronous speed, as well as speeds above and below this speed. A comparison was made with and without a damping controller. When the controllers were optimized for 8 m/s, there exists stability for the synchronous and super synchronous modes, but not sub synchronous. An identical result was seen at 8.5 m/s. However, when the parameters are optimized at 7 m/s, there exists stability across all three operating regions. With regards to efficacy of damping controller, there were scenarios where the controller proved to be effective and scenarios where it failed to produce a superior result. The control method focused more on the effects of using a damping controller and not on the optimization capabilities of the BFO. There was no comparison between optimization using BFO and optimization using another method, like Ziegler Nichols. As a result, the superiority of BFO in this application could not be validated.

PI controllers were optimized using BFO in [48]. The PI controllers were responsible for regulating the rotor currents and dc link voltage of a DFIG. The fitness function used was ITAE. The method was tested under step references and random rotor speed changes and compared to PSO and Genetic Algorithm (GA) based PI controller tuning. Considering the dc link voltage under step reference, the rise time of all three algorithms seems similar. BFO produced the best overshoot, which was 25.33% and 132.8% superior to GA and PSO respectively. The corresponds to a superiority of 17.5% and 253.21% respectively for the settling time. Considering the direct rotor current, BFO yielded the best rise time, marginally beating PSO. However, the PSO optimized controller produced large fluctuations in response. The overshoot superiority was once again exhibited by BFO, this time the result being 54.54% and a huge 834.62% superior to GA and PSO respectively. This corresponds to a superiority of 71.82% and 27.96% respectively for the settling time. For the quadrature rotor current, BFO presented the poorest rise time, but the best overshoot and settling time. The overshoot was superior to GA and PSO by 74.79% and 84.83% respectively and the settling time was superior to GA and PSO by 31.3% and 158.81% respectively. BFOA method also produced a good THD level of the grid current, but this was not compared to the THD produced by the other two control methods.

### 4.3 Grey wolf optimization

Grey wolf optimization (GWO) is a MOT which is based on the behavior of the grey wolf. Proposed by Mirjalili, this algorithm is based on the hunting and democratic conduct of grey wolves [81], [82]. The social hierarchy of a pack of grey wolves is such that the alpha wolf is the highest ranked wolf and serves as the leader of the pack. The beta wolf is responsible for relaying information from the alpha wolf to the other wolves and assists the alpha wolf in decision making. The delta wolf is the third ranked wolf, and their duties include finding paths, killing, and taking care of the other wolves. Finally, all other wolves are classified as omega wolves and obey the rules of the wolves above them [81]. The mathematical representation of a grey wolf surrounding a prey can be represented as [81], [82]:

(9) $$\bar{D}=|\bar{C}.\bar{X_{p}}(t)-\bar{X}(t)|$$

(10) $$\bar{X}(t+1)=\bar{X_{p}}(t)-\bar{A}.\bar{D}$$

Where $\bar{X_{p}}(t)$ is the position of the prey, $\bar{X}(t)$ is the current position of the wolf, $\bar{X}(t+1)$ is the updated position of the wolf and $\bar{A}$ and $\bar{C}$ are co-efficient vectors. $\bar{A}$ is based on the current and maximum iteration numbers, and a random number in the range [0,1]. $\bar{C}$ is only based on a random number in the range [0,1]. This is a different random number from the one used to determine $\bar{A}$ [81], [82], [83]. The value of $\bar{A}$ critically influences the outcome of the algorithm. If |A|<1 then this indicates that the wolf attacks the prey. If |A|>1 it means that the wolf moves away from the prey and attempts to locate a more suitable prey. The randomness of $\bar{C}$ improves the chances of the algorithm to obtain the global optimum solution to the problem [82]. Considering the solution to a given problem, alpha refers to the best solution, beta refers to the second-best solution and delta refers to the third best solution [83]. The position update of a particular wolf can be represented as [81], [82]:

(11) $$\bar{X}(t+1)=\frac{X_{1}+X_{2}+X_{3}}{3}$$

Where $X_{1}$, $X_{2}$ and $X_{3}$ are absolute values which are based on the position of the three best wolves ($X_{\alpha },\bar{X_{\beta }}$ and $X_{\delta }$) respectively, three random number ($A_{1}$, $A_{2}$ and $A_{3}$) respectively and three absolute values ($D_{\alpha },D_{\beta }$ and $D_{\delta }$) respectively. $D_{\alpha }$, $D_{\beta }$ and $D_{\delta }$ are based on $\bar{X_{\alpha }}$, $\bar{X_{\beta }}$ and $\bar{X_{\delta }}$ respectively, as well as the coefficient vector *C* and the current position if the respective wolf. $A_{1}$, $A_{2}$ and $A_{3}$ are determined in the same manner as *A*, noting that a new random number is generated for the estimation of each.

**Figure 11:**
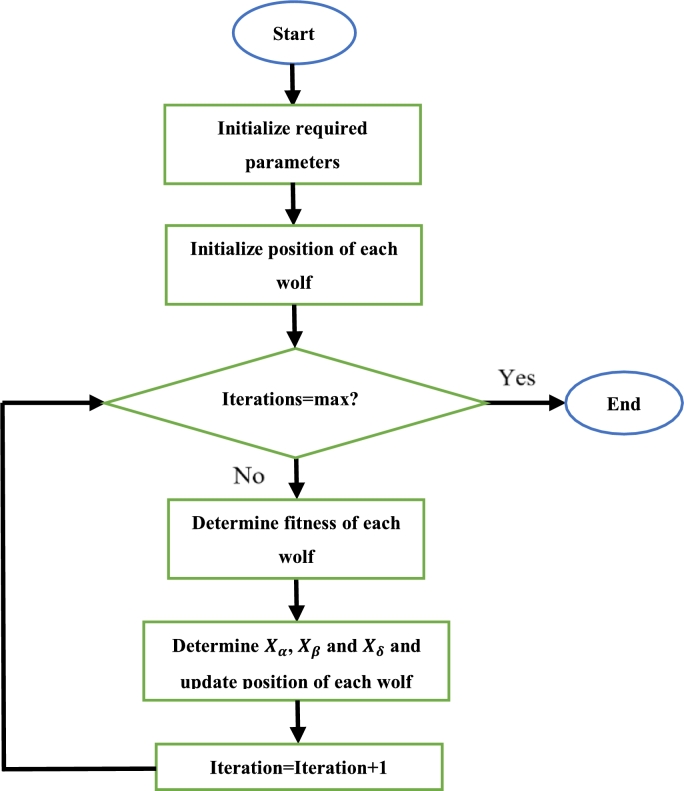
GWO algorithm flowchart [84].

Initially, the required parameters are defined. Then, each wolf is assigned a random position. The fitness of each wolf is then computed. The position of each wolf is then updated according to (10). The fitness of each wolf is evaluated once more. If the new fitness of any wolf is superior to that of the previous fitness function of that same wolf, that wolf updates its fitness value (and hence position). The three best fitness values are chosen, and their corresponding positions are noted. Then, equation (11) is applied to each wolf to update the positions of each wolf once more. Once again, the fitness of the wolves is determined, and the wolf with the best fitness is known to be at the best position. The process is continued until all iterations have been completed, each time updating the position of each wolf if the calculated fitness function value of that wolf is superior to the current best fitness function. After all iterations have been completed, the wolf with the best fitness is taken as the optimal solution [81], [82], [83]. The steps to execute the GWO algorithm can be seen in Fig. 11 [84].

The conventional GWO has the merit of strong local search capabilities [82]. However, its demerits are poor global search capabilities and slow convergence at the latter part of the algorithm [81], [82], [83]. The authors in [82] proposed the use of the quantum behaved search mechanism to enhance the ability of the GWO algorithm to prevent entrapment in the local optimum. It does so by updating the position of each wolf using a probability density function based on Monte Carlo stochastic simulation. This is based on quantum uncertainty. The proposed algorithm was tested on various benchmark functions, both single and multipeak functions. The proposed algorithm was compared to other improved GWO algorithms, as well as the conventional GWO algorithm. For four of the five benchmark functions, the proposed algorithm produced an equivalent best result to one of the modified algorithms. For the remainder benchmark function, the proposed algorithm exhibited dominancy in accuracy and stability over the next best by incomparable margins (above $5e^{5}\text{\%}$). The convergence rate of the proposed algorithm was shown for each benchmark function. However, no comparison of convergence rate between the proposed algorithm and the conventional algorithm was provided. Hence, there exist the probability that in the process of enhancing the algorithms global search ability, the convergence rate of the algorithm was compromised.

The behavior of cats is utilized to modify the GWO algorithm in [83]. In an idle state, cats either seek or track prey. The seeking and tracking behavior is integrated into the social behavior of the grey wolf. In tracking mode, the position of a particular wolf is updated in a manner similar to that of PSO. The updated position is dependent on the current position and the updated velocity. The updated velocity is calculated using the current velocity, the current position of the respective wolf, the current position of the best wolf, and two random numbers in the range [0,1]. The seeking mode utilizes a stochastic change in the dimension of each wolf in order to improve the randomness of the algorithm. Reference [83] combines both the tracking and seeking modes and applied this to the GWO algorithm. The Tracking Grey Wolf Optimization (TGWO), Seeking Grey Wolf Optimization (SGWO) and Tracking-Seeking Grey Wolf Optimization (TS-GWO) algorithms were applied to various benchmark functions and compared to numerous swarm-based MOT, including the conventional GWO. Considering the 30-Dimensional unimodal and multimodal functions, the TGWO and TSGWO combined produced the best average value and standard deviation for 15 of the 16 functions. Only for one of the functions, the SSA yielded the best result. Considering the 100-Dimensional unimodal and multimodal functions, TGWO and TSGWO combined produced the best average value for all the functions. Considering the standard deviation, there existed a couple of scenarios whereby the WOA proved to be dominant. A similar scenario is observed for the fixed dimension multimodal functions, this time the MFO displaying superiority (in both accuracy and stability) for one of the functions. For another function, despite being inferior to TGWO and TSGWO in terms of accuracy, the ALO exhibited stronger stability. The proposed algorithms were also compared to various GWO hybrid algorithms and once again tested on 30-Dimensional and 100-Dimensional unimodal and multimodal functions. Considering the 30-Dimensional unimodal and multimodal functions, TGWO and TSGWO combined generated the best average value and standard deviation four 14 of the 17 functions. A near identical result is observed for the 100-Dimensional unimodal and multimodal functions, this time the proposed algorithm also being inferior in stability to another algorithm for one of the functions. The TGWO and TSGWO generated the best average value in all the fixed dimension multimodal functions, but failed to display stability dominancy in 50% of the cases.

To enhance the convergence rate, a convergence factor was introduced in [81]. This convergence factor modifies the way in which the coefficient vector *A* is estimated. This convergence factor is based on the current and maximum iteration numbers. To improve the global search capability of the algorithm and produce a strong balance between exploitation and exploration, the BFGS algorithm as well as the Levy flight technique were used. Local Diversity Measure and Global Diversity measure were used to determine if the wolves perform local or global search. The local search update is modified using the BFGS algorithm, which is based on the position of the best wolf. The proposed algorithm also makes use of a probability criterion which allows some wolves to update their position using the modified equation and some wolves to update their position using the conventional equation. The global search update is based on the use of the Levy flight technique, which is calculated using the current position of the respective wolf as well as the position of a randomly chosen wolf. The proposed algorithm was tested on a range of unimodal, multimodal, and fixed dimensional multimodal benchmark functions. The proposed algorithm was compared to various swarm-based MOT, including the conventional GWO. For the unimodal functions, the proposed algorithm outperformed all the other algorithms in terms of global search capability and convergence rate (after a maximum of 10 iterations). Considering the unimodal functions, the proposed algorithm displayed the same results that were seen for the unimodal functions, except for one function. In this function, the Imperialist Competitive Algorithm based PSO produced the best global search capability, with the Ant Lion Optimization producing the best convergence rate up to 90% of the maximum number of iterations. Nevertheless, the proposed algorithm produced a better global search capability to the conventional GWO, but the former was inferior to the latter in terms on convergence rate up until 99% of the maximum number of iterations. For the fixed dimensional multimodal functions, the proposed algorithm produces competitive results in terms of global search capability but was inferior to the conventional GWO. The proposed algorithm, however, produced the best convergence rate (after a maximum of 75 iterations). However, for the convergence curves, only limited data was available. It was also observed that throughout the duration of the investigation, there existed various scenarios whereby the proposed algorithm was inferior to other algorithms in terms of stability.

Considering the application of GWO to the DFIG, the authors in [85] applied GWO to fractional order PID (FOPID) control. This is due to the improved closed loop performance and enhanced disturbance rejection capabilities of the FOPID controllers. The FOPID controller makes use of two additional parameters, which ensures that the performance does not degrade if the rotor resistance varies. Since the GWO requires initial solutions to be generated, this method initially tunes the three parameters of the fractional order PID controllers using the Ziegler Nichols method and then proceeds to apply GWO to these parameters. The method was compared to PSO-PID and BFO-PID and was shown to be superior to both these methods with regards to the settling time, as well as the rise time and percentage overshoot. An identical result was observed when the rotor and stator parameters were varied by 25%. The proposed algorithm also produced a better disturbance rejection response than the BFO-PID but was inferior in this aspect to PSO-PID. However, the FOPID controller should have also been optimized using other MOT (such as PSO and BFO), so as to provide an accurate evaluation of the optimization technique and controller combination. A grouped grey wolf optimization strategy is presented in [86] and applied to the optimization of PI controllers for control of the DFIG. The proposed algorithm splits the wolves into two groups. These are the cooperative hunting group, and random scout group. The random scout group searches unknown territory, much like the scout bees in ABC. This is to enhance the exploration capability of the algorithm. In the cooperative hunting group, the number of beta and delta wolves increase to two and three respectively. This is to enhance the exploitation capability of the algorithm. The proposed algorithm was compared to that of GA, PSO, MFO and the conventional GWO. The results showed that optimized of PI controllers via the proposed method yielded a significant reduction in steady state ripple of both active and reactive powers. This result holds true, even when tested under the case of a 30% drop in the grid voltage.

### 4.4 Artificial bee colony

The artificial bee colony (ABC) algorithm is a MOT inspired by on the hunting behavior of honeybees. Created by Karaboga, this algorithm divides the hunting bees into three types; employed bees, onlooker bees and scout bees. An employed bee is a bee which has found an exploitable food source. The onlooker bee awaits the information obtained by the employed bee to decide which food source to visit. The scout bee randomly searches for food on its own [87], [88], [89]. For every food source, there exists one employed bee. When the food source of an employed bee becomes depleted (either by that of another employed bee, or an onlooker bee), it develops into a scout bee [88]. The information regarding the food source is communicated from the employed bees to the onlookers via dancing. The onlookers observe the dance done by the employed bees to choose the best quality food source [89]. The hunting done by the bees is known as foraging and is defined by four characteristics [89]:

*Positive feedback: This refers to a proportional increase in onlookers visiting rich food sources*
*Negative feedback: This refers to the bees eventually ceasing to visit the areas where there exist poor food sources*
*Fluctuations: This refers to the random search behavior by the scouts*
*Multiple interactions: This refers to the exchange of information that exists between the employed bees and onlookers*

At first, food sources are randomly chosen (by scout bees). A scout who discovers a food source becomes an employed bee. The employed bees then search for another food source in the locality of the food source they have found already. They do this by means of visual representation. They then evaluate the quality of this food source and apply the greedy selection. In the greedy selection, if the new food source is of better quality than the previous food source then the previous food source is deleted from the memory of the employed bee and replaced with the new food source. If not, then the old food source remains in the memory of the employed bee. The onlooker bee obtains this information from the employed bee via utilization of the dancing area. The onlooker bees then choose a food source to go to, based on probability and the information received from the employed bees. In such case, the best food source has the best probability of being chosen (using a method such as the roulette wheel). Upon the choosing of a food source by the onlooker bee, the onlooker bee searches for another food source in the same vicinity of the chosen food source. Like the employed bee, the application of the greedy selection is implemented to the previous food source and the newly chosen food source. After a preset number of attempts, if the employed bee's food source fails to improve, they become scout bees and their food sources are discarded. Once again, as in step one, these scout bees randomly search for a new food source. This process is repeated until the satisfaction of a specified termination criteria [89], [90]. The magnitude of employed bees is determined via the number of food source. There exists one food source per bee [88], [89]. A food source represents a possible solution to a problem and the quality of the food source corresponds to the fitness of the solution [89], [90]. The population size consists of several solutions to the problem which can be represented as [89]:

(12) $$x_{i,j}=[x_{1,1},x_{1,2}\ldots x_{S,D}],\;i\in [1,2,3\ldots S]\;\text{and}\;j\in [1,2,3\ldots D]$$

Where *S* is the population size (total number of solutions) and *D* denotes the number of parameters to be optimized. The initial position of an employed bee is shown as [89], [90]:

(13) $$x_{0,i,j}=x_{j\;min}+\Phi_{ij}(x_{j\;max}-x_{j\;min})$$

Where $x_{0,i,j}$ is the position of the $i^{th}$ employed bee in the $j^{th}$ dimension, $x_{j\;max}$ and $x_{j\;min}$ are the upper and lower bounds respectively of the search space in the $j^{th}$ dimension and $\Phi_{ij}$ is a random number in the range [−1,1]. Considering real bees, onlookers and employed bees modify their solution based on visual representation. In the case of artificial bee colony, these bees randomly choose a new food source and compare the richness of this food source with the one in their memory. If the new food source is richer than the one in their memory, the memory is updated [56]. The probability of an onlooker bee choosing a particular food source is given by [90]:

(14) $$p_{i}=\frac{f_{i}}{\left(\sum_{n=1}^{s}f_{i}\right)}$$

Where $p_{i}$ is the probability of the $i^{th}$ solution and $f_{i}$ is the fitness value of the $i^{th}$ solution. Note that this corresponds to a larger fitness function being better, so if the objective is to minimize the fitness function, then the calculated value needs to be inverted before using the above equation. To produce a new solution from the old solution, the following is used [90]:

(15) $$v_{ij}=x_{ij}+\Phi_{ij}(x_{ij}-x_{kj})$$

Where $v_{ij}$ is the new solution, $x_{ij}$ is the previous solution of the bee (either employed bee or onlooker bee), k∈[1,2…S], j∈[1,2…D] and $\Phi_{ij}$ is a random number which lies in the range [−1,1]. Note that *k* and *j* are chosen randomly, but *k* needs to be different from *i*.

**Figure 12:**
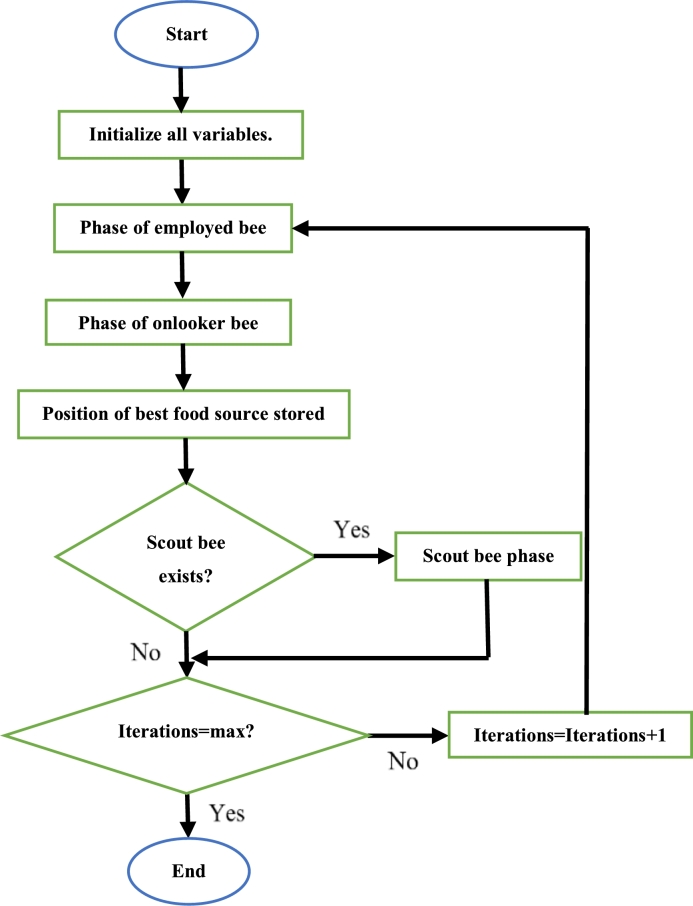
ABC algorithm flowchart [92].

Initially, the required parameters are defined. Then the number of employed bees are defined, and their positions initialized according to (13). The fitness of each employed bee is then computed and is used to select the probability of an onlooker bee moving to the position of a specific employed bee. This is seen in (14). Once the onlooker bee moves to the position of an employed bee, it updates its position according to (15). The cost function value of the onlooker bee at the newly discovered position is then evaluated. If the cost function value of the new position is superior to that at the previous position, the onlooker bee takes on this new fitness function value (hence new position). The employed bee also updates its position according to (15) and their fitness function value at this new position is computed. As like the onlooker bee, if the fitness function value at this new position is superior to that at the previous position, the employed bee takes on this new fitness function value (hence position). Once again, the onlooker bee choses a position based on the fitness values of the employed bee. If the positions of the employed and onlooker bees cannot be improved after a certain number of search attempts, they are converted into scout bees and are assigned random positions. This continues until all iterations have been completed. Once this is so, the bee with the best fitness value is at the optimal position [91]. The steps to execute the ABC algorithm can be seen in Fig. 12 [92].

The conventional ABC algorithm has the merit of a strong global search capability [93] but suffers the demerit of a slow convergence [94], [95], [96]. The authors in [97] proposed a new method to update the position of a bee. The modified position update equation is a function of the global best, the position of a random bee and a random number in the range [−1,1]. The proposed method was applied to various benchmark knapsack problems and compared to the conventional ABC algorithm. Considering the average value, the proposed algorithm produced a superior result for three of the problems. The greatest difference being 3.11% and the smallest difference being 0.02%. For the remainder functions, the proposed technique was equivalent to the conventional algorithm. It was also observed that in numerous cases, the proposed algorithm exhibited a superior stability. Concerning the convergence rate, the proposed algorithm yielded superior results in four cases, the smallest superiority being 61.68%. The authors in [95] propose a new method to update the position of the various bees. This is based on the use of the bee's current position, two cumulative fitness values of all the bees, the position of the best bee and two random numbers in the range [0,1]. The method of position updating does not apply to the worst employed bee. The position of the worst employed bee is updated using the current and previous position of the best bee, an integer which is either 0 or 1 and two random numbers in the range [0,1]. The position of the onlooker bee is updated using the cumulative fitness of all the bees, the total number of bees and the position of any onlooker bee whose position is superior to that of the bee being updated. The proposed method was applied to various benchmark functions, as well as two industrial problems. The proposed method was compared to various swarm-based MOT including the conventional ABC algorithm. However, the results in the paper were inconclusive hence the proposed method could not be validated.

The authors in [96] made use of two unique equations for updating the position of the employed and onlooker bees. The position update of the employed bee is based on the current position of the bee and the position of a random bee. The position update of the onlooker bee is based on the current position of the bee, the position of the best bee and the position of three bees chosen at random. The proposed algorithm was tested on a synthetic web service selection problem and compared to various swarm-based MOT, including the conventional ABC algorithm. The proposed method proved to have a superior convergence rate to the other algorithms, after 10 iterations. In terms of reliability, the proposed technique exhibits dominancy after completion of 30% of the maximum number of iterations.

In [98], ABC is utilized in the control of the DFIG. The authors in [98] used ABC to optimize the parameters of the field-oriented control PI controllers. Two cases are presented in this paper. In the first case, the ABC algorithm was applied to only the RSC using a fitness function based on the stator active power, stator voltage and current regulation errors. Three weighting factors are used in this fitness function, which were chosen by the ABC algorithm. In the second case, the ABC algorithm was applied to both the RSC and GSC. The fitness function used was based on the stator active power, stator voltage, current regulation, grid current and dc link voltage errors. This time, five weighting factors were used and chosen by the ABC algorithm. In total, 10 PI controller gains were optimized. The control method was applied to a 9 MW DFIG and compared to the GWO method of PI gain optimization, as well as the traditional PI controller optimization method. When only the RSC gains were considered, it is observed that ABC yielded the best overshoot value, being superior to GWO and the advisory method by more than $2e^{3}\text{\%}$ and $3e^{3}\text{\%}$ respectively. A similar observation is made for the settling time, this time the superiority being 0.52% and 0.78% respectively. Considering the rise time, the ABC exhibited dominancy to the other techniques by a magnitude greater than 100% When the RSC and GSC gains were considered, it was seen that ABC yielded the best overshoot value, being superior to GWO and the advisory method by 97.7% and 169.73% respectively. A similar observation is made for the settling time, this time the superiority being 0.21% and 0.26% respectively. Considering the rise time, the ABC exhibited dominancy to GWO by 0.32%, and to the supervisory method by more than 100%.

### 4.5 Whale optimization algorithm

Whale optimization algorithm (WOA) is inspired by the hunting tactic of the humpback whale. The hunting strategy of the humpback whale is separated into three parts: searching, encircling and bubble-net attacking [99], [100], [101]. During searching, the humpback whales exchange information about prey to each other. This is to ensure that all the whales stay close to the prey. Consider the following [102], [103]:

(16) $$X_{i}(t)=[X_{i,1}(t),X_{i,2}(t)\ldots X_{i,D}(t)]$$

Where $X_{i}(t)$ is the current position of the $i^{th}$ whale and *D* is the number of search space dimensions. The position of the whales at the next sampling instant can be updated using three methods. The first method is via a random search and is shown as [99], [102], [103]:

(17) $$X_{i}(t+1)=X_{r}(t)-A|{C\times X}_{r}(t)-X_{i}(t)|$$

Where $X_{r}(t)$ is the position of a whale chosen at random and *A* and *C* are coefficients. *A* is based on the current and maximum iteration numbers, as well as a random number in the range [0,1]. *C* is based only on a random number in the range [0,1]. It is important to note that the random numbers used in the evaluation of *A* and *C* are generated independently. The second method is to encircle the prey. To encircle the prey, each of the whales update their positions based on the best position found thus far. This update is represented as follows [99]:

(18) $$X_{i}(t+1)=X_{p}(t)-A|C\times X_{p}(t)-X_{i}(t)|$$

Where $X_{p}(t)$ is the best position found thus far (at iteration *t*). The third method is via the use of bubble net attacking. Bubble net attacking is a mathematical model used to imitate the spiral movement of the humpback whale [67], [68]. In bubble net attacking, the whales update their positions as follows [99], [102], [103]:

(19) $$X_{i}(t+1)=X_{p}(t)-|X_{p}(t)-X_{i}(t)|e^{bl}\times \cos(2\pi l)$$

Where *b* is a limited constant and *l* is a random number in the range [−1,1]. The method of position updating to be used is based on a random number *q* in the range [0,1], as well as the value of *A*. If *q* is less than 0.5 and the magnitude of *A* is greater than one, the whale positions are updated using encircling of the prey. If *q* is greater than 0.5 and the magnitude of *A* is greater than or equal to 1, the whale positions are updated randomly. Else, the bubble net attacking method of position updating is used [99].

**Figure 13:**
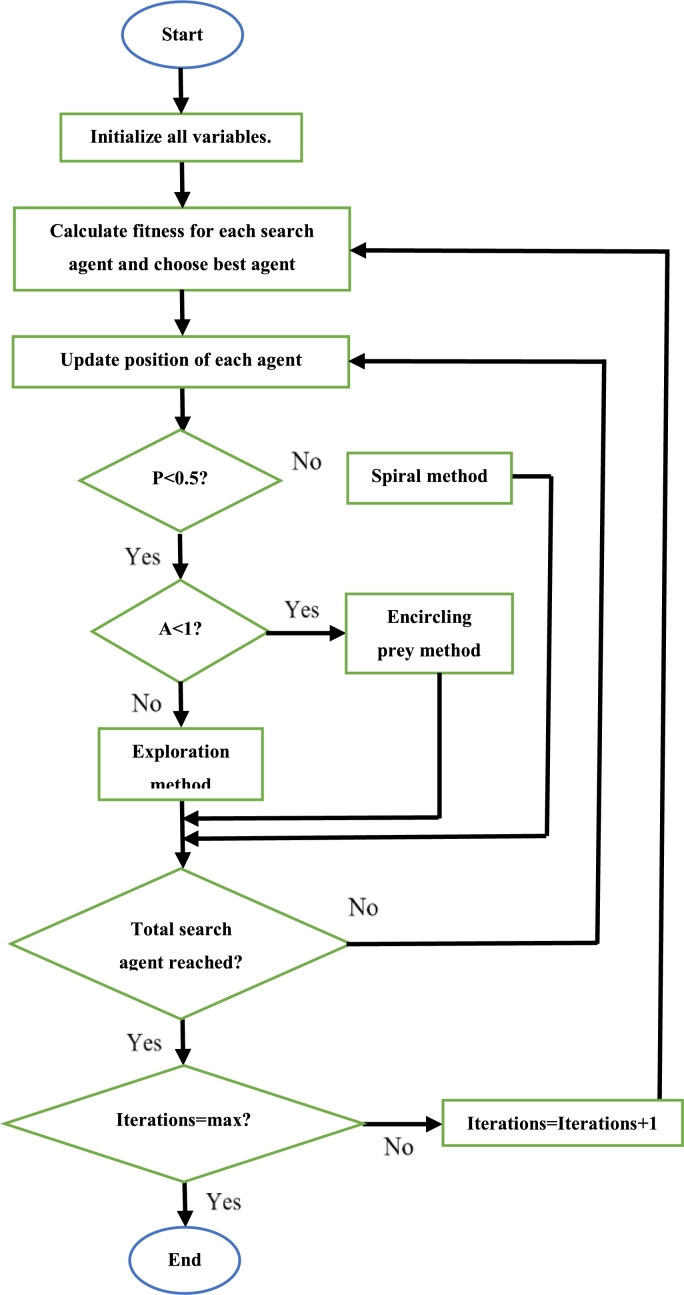
WOA flowchart [104].

Initially, the required parameters are defined. Then, each whale is given a random position. The fitness of each whale is calculated and the whale with the best fitness value is noted. The random numbers *P* and *A* are then generated. If the magnitude of *A* is less than 1 then the position of each whale is updated using (19). If *P* is less than 0.5 and the magnitude of *A* is greater than one, then the position of each whale is updated using (18). Lastly, if *q* is greater than or equal to 0.5, the position of each whale is updated using (17). After the update is completed, the fitness of each whale is calculated and replaces the current best fitness value (of that whale) if its value is superior to that of the current best. This continues until all iterations have been completed. Once this is so, the whale with the best fitness is said to be at the most optimal position [99]. The steps to execute the WOA can be seen in Fig. 13 [104].

Although the WOA has the merit of being able to evade the local optima hence obtain the global solution [105], it suffers the demerits of a slow convergence speed and low accuracy [106]. The authors in [107] proposed the use of a new method to update the position of the whales. This is based on the golden sine operator and makes use of the current position of the whale, as well as two random numbers lying in the range [0,1]. The proposed algorithm was tested on a range of unimodal, multimodal, and combined functions and compared to various other MOT, including the conventional WOA. Considering the unimodal functions, the proposed algorithm generated the best average value and standard deviation in 5 of the 7 cases. For the other two cases, the algorithm is inferior to PSO in both accuracy and stability. For the multimodal functions, the proposed algorithm displayed inferiority in both average value and standard deviation to the Firefly Algorithm for two functions. For both functions, for both the average value and standard deviation, this inferiority was approximately 100%. For the fixed dimensional functions, the proposed technique did not do well, being inferior to various algorithms in majority of the cases. This was in terms of both accuracy and stability. In cases where the proposed technique produced the dominant average value, this occurred after a maximum of 22% of the total number of iterations occurred.

The authors in [108] also proposed a new method to update the position of the whales. This is done via a chaotic map and nonlinear inertial weights. The method is complex and involves a significant number of numerical computations. The proposed algorithm was tested on various benchmark functions, at 100, 500 and 1000 dimensions. For all the investigated scenarios, the proposed techniques exhibited superior performance in both accuracy and stability. Considering convergence, for the 100 and 500 dimension sets the proposed algorithm only displayed clear dominancy after completion of 70% and 80% respectively of the maximum number of iterations. For the 1000 dimension set, this value reduces drastically to 30%, indicating the efficacy of the proposed solution when attempting to optimize large scale global problems.

An improved Bernoulli shift map was introduced in [109] to initialize the population of whales so to enhance the algorithm global search ability. A modified Levy flight based position update equation is also proposed to enhance the global search capabilities of the algorithm. The method also optimizes the factor of convergence (a) to enhance the algorithm rate of convergence. The modified convergence factor is based on the value of the current and maximum iteration numbers, the best and worst fitness of that particular whale thus far and a random number in the range [1,2]. The method proved to improve the algorithms search accuracy and rate of convergence. The proposed algorithm was tested on numerous benchmark functions and was compared to various MOT, including the conventional WOA. When considering search accuracy, it was observed that the proposed algorithm was inferior to the Enhanced advanced guided differential evolution algorithm and SHADE algorithm in only three of the twenty functions. In scenarios where the proposed algorithm produced the best average value, this occurred after a maximum of 40% of the total number of iterations has occurred.

### 4.6 Crow search algorithm

Proposed by Askarzadeh, the idea behind the crow search algorithm (CSA) rests on the hiding of food of crows. Crows are highly intelligent birds, who can remember faces and remember the location of their stored food. The most important aspect of this algorithm is that when a crow attempts to retrieve its stored food, another crow may follow it and steal the food [44], [110]. Initially, the crow has a random position which it stores in its memory. The $i^{th}$ crow then follows the $j^{th}$ crow to attempt to steal its food. There exist two scenarios; either the $j^{th}$ is aware that the $i^{th}$ crow is following it, or it is not aware of this. If it is aware, it would attempt to trick the $i^{th}$ crow by flying to a random location. If not, then the $i^{th}$ crow is successful in stealing the food of the $j^{th}$ crow. This can be represented as follows [44], [110], [111]:

(20) $$x_{i}(t+1)=f(x)=\left\{ \begin{array}{cc} x_{i}(t)+r_{i}\times {fl}_{i}(t)\times (m_{j}(t)-x_{i}(t)),\; & r_{j}\geq {AP}_{j}^{t}\\ \text{some random position},\; & \text{otherwise} \end{array}\right.$$

Where $x_{i}(t+1)$ is the updated position of the $i^{th}$ crow, $x_{i}(t)$ is the current position of the $i^{th}$ crow, $fl_{i}(t)$ is the length of flight of the $i^{th}$ crow and be taken as a randomized number between 1 and 2, $m_{j}(t)$ is the location of the food of the $j^{th}$ crow (taken as the current position of a randomly chosen crow), $r_{j}$ is a randomized number between 0 and 1 and $AP_{j}^{t}$ is the probability of awareness of the $j^{th}$ crow to the intention of the $i^{th}$ crow. It is a random number between 0 and 1. If the fitness function value of the $i^{th}$ crow at the new position is superior to that stored in its memory, the $i^{th}$ crow updates its memory. Else it disregards the new solution [44], [110], [111].

**Figure 14:**
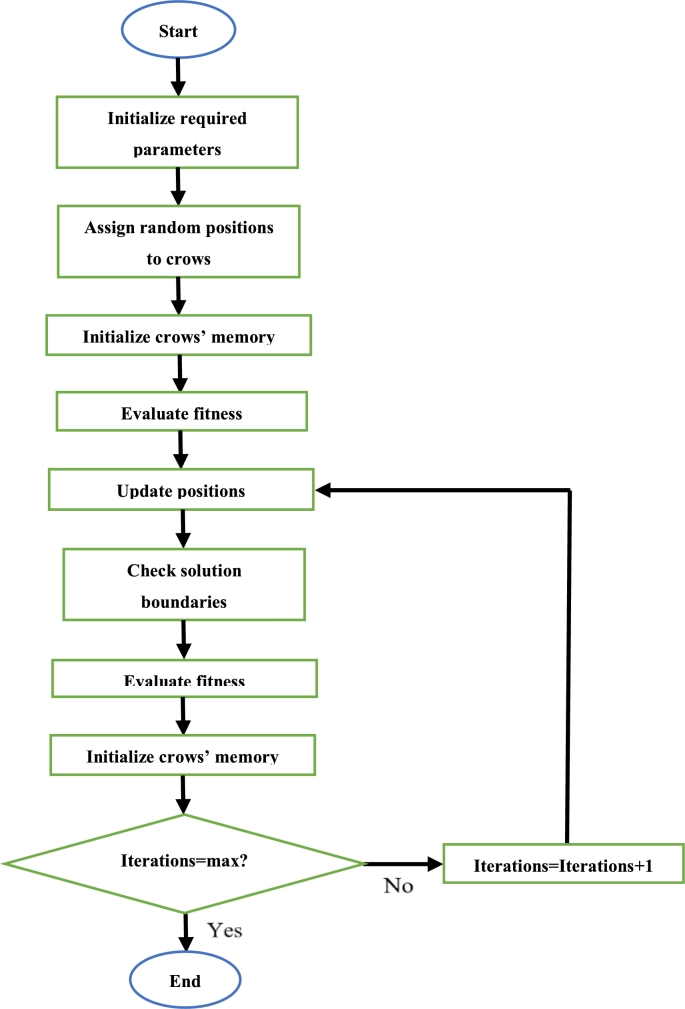
CSA flowchart [44].

Initially, the required parameters are defined. Then, each crow is given a randomized position. The position of each crow is then updated by using (20). Afterwards, the fitness of each crow is calculated and the best position which exists in each crows' memory is updated if the new fitness value is superior to the fitness of the position in its memory. This continues until all iterations have been completed. Once this is so, the crow with the best position is said to be the best solution [44]. The steps to execute the CSA can be seen in Fig. 14 [44].

The merit of the conventional CSA is that it is a flexible algorithm which requires knowledge of only a few parameters [44]. However, the demerits of this algorithm are a slow rate of convergence and being easily entrapped in the local optima [110]. The authors in [110] proposed various modifications to the conventional CSA. In the first modification, the existing position update equation is multiplied by a weighting factor which is based on the current iteration and maximum iteration numbers. In the second modification, the initial positions are generated via a spiral search. In the third modification, another position update equation based on a Gaussian mutation, is proposed. The first modification is proposed to improve the algorithms rate of convergence, whereas the second and third modification is proposed to enhance the algorithms global search ability and prevent it from being entrapped into local optima. The proposed algorithm was tested on various fixed dimensional, multimodal, and unimodal benchmark functions and compared to various other swarm-based MOT, including the conventional CSA. The proposed algorithm was evaluated at 30, 50 and 100 dimensions. For the 30 dimensional set unimodal functions, the proposed algorithm yielded the best result (in terms of both accuracy and stability in 6 of the 7 functions). Only for one function was the proposed algorithm inferior to the Harris Hawks Optimization technique. The inferiority to the best result, in terms of average value and standard deviation, was 93.56% and 94.69% respectively. For the 30 dimensional set multimodal functions, a similar result is observed. This time, the proposed technique being inferior to GA, with this inferiority being at 100% for both the average value and standard deviation. A similar trend is displayed for the 50 and 100 dimensional sets, with the proposed algorithm only being inferior in one instance of each case. There is, however, a reduction in inferiority for the 50 and 100 dimensional set multimodal functions. This points to the possibility of the algorithm exhibiting dominancy when optimizing large dimensional problems. Considering the fixed dimensional functions, the proposed algorithm yielded the best result in 8 of the 10 cases, being inferior to the GWO algorithm and the Butterfly Optimization Algorithm.

The convergence rate of the proposed algorithm was only displayed for three of the unimodal and multimodal functions and two fixed dimension functions. Despite the superior average value and standard deviation of the proposed algorithm, it was observed that in various instances, the proposed technique required more than 100 iterations to exhibit dominancy. In some cases, more than 1500 iterations were required. This points to a slow convergence, and should be modified for the algorithm to be considered competitive.

The CSA was applied to the PI control of a DFIG in [112]. Only the RSC was considered, and three PI controllers were tuned. The fitness function used was ISAE. The control method was tested at both fixed speeds and variable speeds and compared to the conventional method of PI controller tuning, as well as the Genetic Algorithm (GA). Considering the fixed speed operation, it is noticed that the GA produced the best stator active power percentage overshoot and rise time. CSA produced a better percentage overshoot to the conventional method, but an inferior rise time. For the stator reactive power, once again GA produced the best percentage overshoot followed by CSA and then the conventional method. The rise time of the conventional method was the best, followed by CSA and then GA. For the dc link voltage, the conventional method produced the best percentage overshoot followed by GA and then CSA. The conventional method also produced the best rise time, with CSA producing the worst in this aspect. Considering the variable speed operation, it is observed that the GA and CSA jointly produced the best stator active power percentage overshoot, with the former producing the best rise time and the latter producing the worst. For the stator reactive power, GA produced the best percentage overshoot, followed by CSA. The conventional method produced the best rise time, with the CSA producing the worst result in this aspect. For the dc link voltage, GA produced the best percentage overshoot and CSA the worst. The conventional method produced the best rise time, followed by CSA.

### 4.7 Bat algorithm

Developed by Xin-She Yang in 2010, the bat algorithm (BA) is inspired by and based on the use of echolocation by microbats. Bats use echolocation to perform various functions, such as locating prey, avoided obstacles, and finding other bats. Echolocation varies greatly, and depends on factors such as frequency, wavelength, loudness, and rate of sonic pulses. The bat algorithm uses a few assumptions of the echolocation used by bats [113], [114]. The first assumption is that every bat utilizes echolocation to determine distance and are able to distinguish between prey and objects. The second assumption is that bats fly randomly at a certain velocity and are also able to vary the wavelength of pulses as well as the pulse rate. The final assumption is that the loudness changes between a specified maximum and minimum. The position of each bat is updated as follows [113], [115]:

(21) $$v_{i}(t+1)=v_{i}(t)+f_{i}(x_{i}(t)-x_{g})$$

(22) $$x_{i}(t+1)=x_{i}(t)+v_{i}(t+1)$$

Where $v_{i}(t)$ is the current velocity of the $i^{th}$ bat, $v_{i}(t+1)$ is the updated velocity of the $i^{th}$ bat, $x_{i}(t)$ is the current position of the $i^{th}$ bat, $x_{i}(t+1)$ is the updated position of the $i^{th}$ bat, $x_{g}$ is the global best position, $f_{i}$ is the frequency of the $i^{th}$ bat. This frequency is calculated using a specified maximum and minimum frequency and a randomized number in the range [0,1]. A randomized number between 0 and 1 is generated and compared to the pulse emission rate of the $i^{th}$ bat. The pulse emission rate is based on the current iteration number and decreases exponentially from the initial specified pulse emission rate. If the random number is greater, the position of the best bat is updated as follows [113], [114], [115], [116]:

(23) $$x_{new}=x_{old}+\in A_{i}^{t}$$

**Figure 15:**
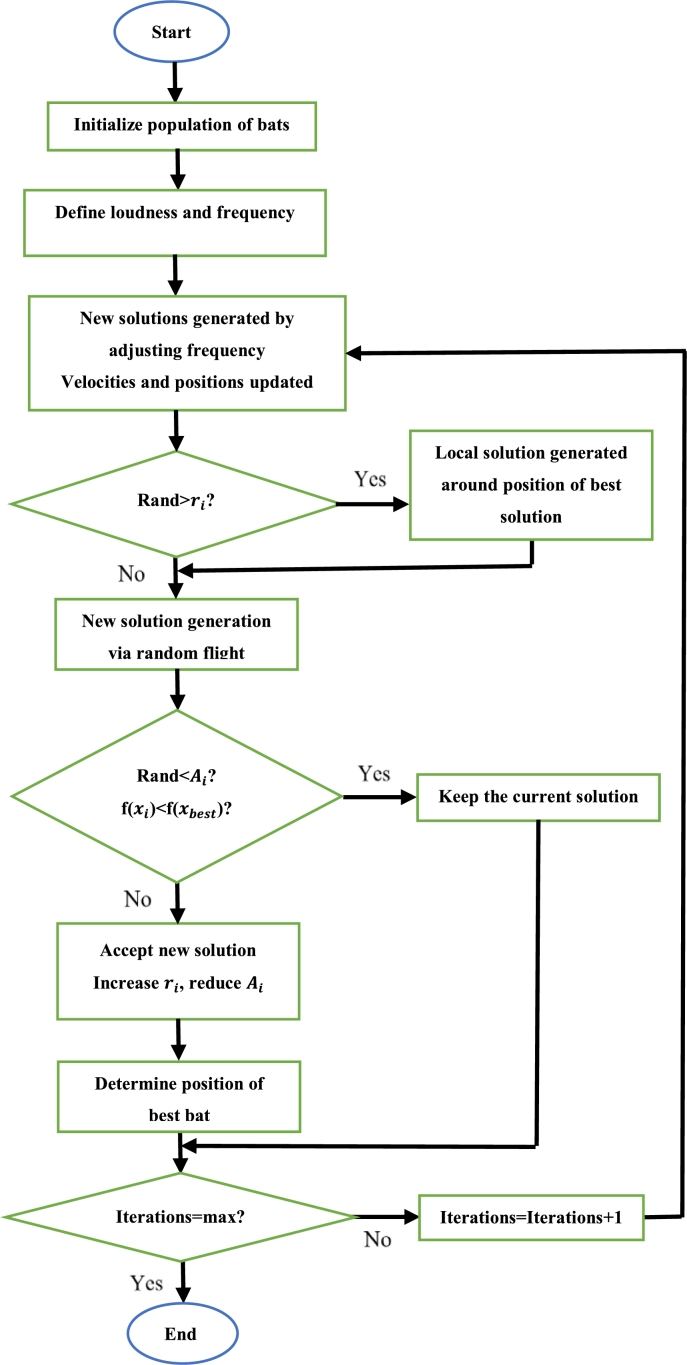
BA flowchart [117].

Where ∈ is a randomized number in the range [0,1], $A_{i}^{t}$ is the current loudness of the $i^{th}$ bat and is based on the current iteration number. Initially, the required parameters are defined. Then, each bat is assigned a random position in the search space. The fitness of each bat is then computed and the position of the bat with the best fitness value is noted. Thereafter, the position of each bat is updated according to (22). The fitness of each bat is computed and if the new fitness value of a bat is superior to the previous fitness value of that same bat, that bat takes on the new fitness value (hence position). Afterwards, a randomized number is defined and if this value is greater than the pulse emission rate of a specific bat, then the position of that bat is updated using (23). The fitness of each bat is evaluated once more. The randomized number is then compared to the loudness of each bat. If the randomized number is less than the corresponding loudness and the fitness value is superior to the previous fitness value, then the bat takes this new position. Else it remains in its previous position. This continues until all iterations have been completed. Once this is so, the bat with the best fitness value is said to be the best solution [113]. The steps to execute the BA can be seen in Fig. 15 [117].

Although the BA has the merit of a fast convergence rate [118], it suffers the demerits of a poor search accuracy and being easily trapped in local minima [114]. The authors in [114] proposed five unique factors of convergence to enhance the global search capability of the algorithm. These convergence factors are of cosine form, sine form, tangent form, power function form and exponential form. All five convergence factors are based on the current and maximum iteration numbers. To enhance the accuracy of the local search, a Gaussian function is introduced. Furthermore, to improve the local search accuracy of the algorithm, a technique based on the enclosing behavior of the WOA, and sine cosine algorithm is applied to the bat algorithm. This is based on the current and maximum iteration numbers, a randomized number in the range [0,2], a randomized number which lies in the range [0,2π] and the values of *A* and *D* obtainable from the WOA. To assess the global search capability of the proposed algorithms, the five convergence factors were applied to various fixed dimension, multimodal and unimodal benchmark functions and compared to the conventional BA. It was observed that for all the unimodal and multimodal functions, the proposed convergence factors produced an equal performance which was superior to that of the conventional BA. For the fixed dimension functions, the proposed algorithms were again superior to the conventional bat algorithm and although they were highly similar to each other in performance, there existed a minute difference. To assess the accuracy of the proposed algorithms, seven datasets were used. The proposed algorithms were once again compared to the conventional BA. On the iris dataset, the power function form produced the best result. On the wine dataset, the tan form was superior. On the BUPA dataset, the exponential form was placed first. On the seed dataset, the sine form produced the best accuracy.

Considering the heart stat log dataset, the Gaussian function produced the best result. On the WDBC and cancer datasets, the exponential form proved to be the optimal algorithm. The authors in [119] integrate the artificial ABC algorithm into the BA to enhance the local search accuracy of the latter. In this method, the bat's position is updated as usually done. Then, a randomized number is created. If the created randomized number is greater than the value of the pulse emission rate, the position of the bat is further modified again. The proposed method was employed to optimize the path of an automatic ariel vehicle and compared to various other MOT, including the conventional BA. The optimization results obtained were superior to that of the conventional BA, as well as various other MOT. However, the algorithms rate of convergence is 50% poorer than the conventional BA. The scholars in [120] attempted to enhance the local search capability of the algorithm, as well as prevent premature convergence. The proposed method comprises of the application of various strategies to the conventional bat algorithm. These are the iterative local search, non-dominant, balance and stochastic inertia weight strategies. Stochastic inertia weight strategy applies a weighting factor to the velocity update equation. This is to enhance the algorithms rate of convergence as well as improve accuracy. The iterative local search strategy applies a specific condition in order to maximize the probability of obtaining the global best. The balance strategy attempts to provide a sense of balance between the global and local search. Since it is impossible to optimize various parameters simultaneously, the non-dominant sorting strategy gives precedence to the solution with the best fitness function. The proposed algorithm is employed on the optimal distribution of flexible fault current limiters and applied to the revised IEEE 33-BUS distribution systems with distributed generation and IEEE 30-BUS benchmark system. The proposed method produced optimal configuration of the system and displayed an improved accuracy when compared to a non-dominated sorting genetic algorithm, as well as a Multi Objective Particle Swarm Optimization which are shown in [121] and [122] respectively. However, the algorithm was not compared to the conventional BA.

A hybrid SMC and BA was used to control a DFIG in [123]. The control method made use of rotor current control to provide stator power control. The fitness function used was the mean square error. The rotor speed was held constant throughout the experiment, but the stator active power reference was stepped up. The stator reactive power was constant at 0. The proposed hybrid controller was compared to the conventional sliding mode controller and conventional PI controller. For the stator active power, stator reactive power and dc link voltage, the proposed controller produced a superior steady state ripple, and a competitive dynamic response. With regards to the percentage overshoot for the stator active power, the proposed controller was superior to the conventional PI controller but came second to the conventional sliding mode controller. This inferiority was calculated to be equal to 36%. It was also observed that there existed a minor unbalance in the stator current waveforms. However, the stator current waveforms for the conventional sliding mode controller and conventional PI controller were not provided, hence the superior or inferior quality of the stator current waveforms from the proposed controller could not be validated.

### 4.8 Squirrel search algorithm

Proposed by Jain et al. in 2018, squirrel search algorithm (SSA) is based on the method of movement and scavenging conduct of flying squirrels. The squirrels usually glide into trees, where they feed and collect nuts. The squirrel search algorithm is based on a few assumptions [124], [125]. The first assumption is that there exists one tree for every squirrel. The second assumption is that there exists one hickory nut tree, a few acorn trees and the rest are normal trees. Hickory nut trees are the best food supply, and the acorn trees are the second-best food supplies. The normal trees are said to contain no food. The number of acorn trees is usually taken as 3. The final assumption is that each squirrel individually attempts to locate food and makes use of the food supplies that are available. If, in a forest there exists *n* squirrels, the position of the $i^{th}$ squirrel (on the $i^{th}$ tree) can be described as [125]:

(24) $$x_{i}=(x_{1,1}.x_{1,2}\ldots x_{n,d}),\;i\in (1,2,\ldots ,n)$$

Where $x_{i}$ is the position of the $i^{th}$ squirrel and *d* is the dimension of the $i^{th}$ squirrel. The initial position of the $i^{th}$ squirrel is [124]:

(25) $$x_{i}=x_{sl}+U\times (x_{su}-x_{sl})$$

Where $x_{sl}$ and $x_{su}$ are the lower and upper bounds of the search space respectively and *U* is a randomized number which lies in the range [0,1]. The fitness value for each squirrel is determined and the squirrel with the best fitness function is said to be in the hickory nut tree. The acorn trees are occupied by the squirrels with the next best three fitness values. The rest are normal trees. From this, in order to feed, some squirrels would move from the normal to the hickory nut tree. The rest of the squirrels would move to the acorn trees. The squirrels on the acorn trees would move to the hickory nut tree. However, if there exists the presence of a predator, the squirrels cannot glide to a food tree, and must move to a random location rather [124].

A squirrel at an acorn tree may move to a hickory nut tree. This is shown as [124]:

(26) $$x_{sa}(t+1)=\left\{ \begin{array}{cc} x_{sa}(t)+D_{G}\times G\times (x_{sh}(t)-x_{sa}(t))\; & \text{if }R_{1}>P\\ \text{A random location}\; & \text{otherwise} \end{array}\right.$$

A squirrel at a normal tree may move to an acorn tree. This is shown as [124]:

(27) $$x_{sn}(t+1)=\left\{ \begin{array}{cc} x_{sn}(t)+D_{G}\times G\times (x_{sa}(t)-x_{sn}(t))\; & \text{if }R_{2}>P\\ \text{A random location}\; & \text{otherwise} \end{array}\right.$$

A squirrel which is at a normal tree may go directly to a hickory nut tree. This is shown as [124]:

(28) $$x_{sn}(t+1)=\left\{ \begin{array}{cc} x_{sn}(t)+D_{G}\times G\times (h(t)-x_{sn}(t))\; & \text{if }R_{3}>P\\ \text{A random location}\; & \text{otherwise} \end{array}\right.$$

Where $x_{sa}(t)$ is the current position of a squirrel in an acorn tree, $x_{sa}(t+1)$ is the updated position of a squirrel in an acorn tree, $x_{sn}(t)$ is the current position of a squirrel in a normal tree, $x_{sn}(t+1)$ is the current position of a squirrel in a normal tree, $x_{sh}(t)$ is the current position of the squirrel in the hickory nut tree. $R_{1}$, $R_{2}$, $R_{3}$ are randomized numbers in the range [0,1], *P* is the probability of predator presence, *G* is the constant of gliding and $D_{G}$ is a gliding distance of random nature. $D_{G}$ is based on various factors. These factors are the air density (usually $1.204\;\text{kg}.{\text{m}}^{-3}$), the gliding velocity (usually $5.25\;\text{m}.{\text{s}}^{-1}$), the surface area of the body of the squirrel (usually $154\;{\text{cm}}^{2}$), the friction coefficient (usually 0.6) and a randomized number which lies in the range [0.675,1.5]. To allow for avoidance of local optima entrapment, the squirrel search algorithm makes use of a seasonal change. Initially, all squirrels are said to be in winter. When a certain criterion is met, the season changes from winter to summer. Two variables are defined. The first variable, $S_{c}^{t}$ is calculated based on the squirrel position in the hickory nut tree as well as the position of a squirrel in an acorn tree. The second parameter, $S_{min}$ is calculated based on the current and maximum iteration number [124]. If $S_{c}^{t}<S_{min}$ then squirrels gliding from the normal trees to the acorn trees update their position according to [124]:

(29) $${x_{si}}^{new}=x_{SL}+Levy(x)\times (x_{SU}-x_{SL})$$

Where ${x_{si}}^{new}$ is the new position of the squirrel gliding from the normal tree to the acorn tree. Levy(x) is based on two individually generally randomized numbers which lie in the range [0,1], as well as a positive constant whose value is less than 2 (generally taken as 0.5) [124].

**Figure 16:**
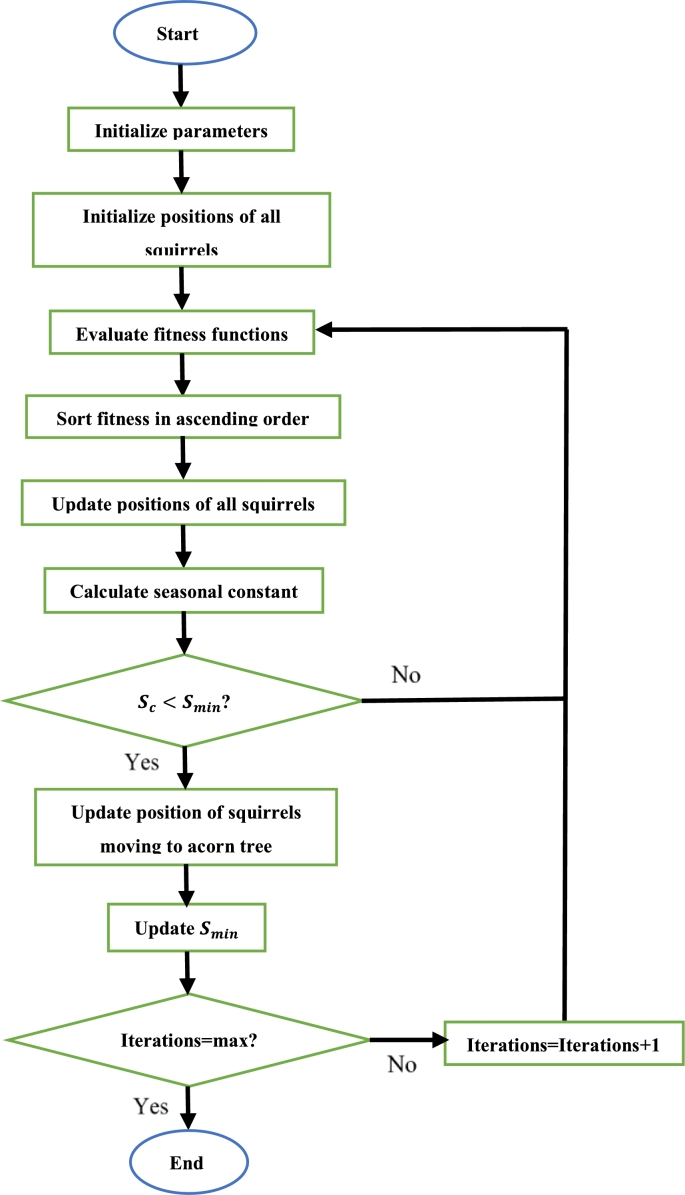
SSA flowchart [124].

Initially, the required parameters are defined. Then, each squirrel is given a random position according to (25). The fitness of each squirrel is then estimated. The squirrel with the best fitness is considered to be in the hickory nut tree while the next few best squirrels (with the next few best fitness values) are considered to be in the acorn trees. The rest are considered to be in the normal trees. From this, the positions of all the squirrels are updated according to (26)–(28). The fitness of each squirrel is computed again and if the current fitness value is superior to the previous fitness value, the squirrel takes on the new fitness function value (hence new position). Once this is done, the seasonal change condition is checked and the position of the squirrels which are gliding from the normal tree to the acorn trees are updated using (29). Once again, the fitness for all squirrels is estimated, and the fitness value (and position) of each squirrel is updated if the new value is superior to the previous value. The squirrel in the hickory nut tree, as well as the squirrels in the acorn trees, are determined. This continues until all iterations have been completed. Once this is so, the squirrel with the best fitness is considered to be the best solution [124]. The steps to execute the SSA can be seen in Fig. 16 [124].

Although the conventional SSA has the merit of a strong stability, it suffers the demerits of a low search accuracy and being easily trapped in the local optima [124]. The authors in [124] attempted to overcome this disadvantage by incorporating the reproductive behavior of the invasive weed optimization algorithm into the conventional SSA. The method generates squirrel offspring via the Gaussian distribution and randomly places these offspring across the search space. The number of offspring produced is proportional to the cost function value of each squirrel and varies linearly with such. Secondly, an adaptive step size strategy is implemented to balance the algorithm exploitation and exploration capability. The proposed algorithm was applied to various fixed dimension, multimodal and unimodal functions and compared to other swarm-based MOT, including the conventional SSA. Of the six unimodal functions, eight multimodal functions and eight fixed dimensional functions, the proposed algorithm only to generate the best average value in one of each set of functions. This inferiority to the best value is 9.23%, 49.85% and 4.92% for the unimodal, multimodal and fixed dimension function respectively. However, considering the standard deviation, there existed various scenarios whereby the proposed algorithm did not yield the best value, in some cases producing the worse result. The points to a lack of stability of the proposed technique. Considering the convergence, it was observed that the proposed algorithm required 75% of the maximum number of iterations in order to exhibit superiority. This amounted to approximately 750 iterations, and therefore indicates a poor convergence.

The scholars in [126] applied the same improved SSA in [124] to the maximum likelihood method for array signal processing based direction of arrival. The method was compared to various swarm-based and evolutionary based MOT, including the conventional SSA. Compared to these techniques, the improved SSA displayed a faster convergence speed, better search accuracy and reduced computational complexity.

### 4.9 Moth flame optimization

Proposed by Miralji in 2015, the moth flame optimization (MFO) algorithm is based on the technique of navigation used by moths. This method of navigation is known as transverse orientation. To ensure a straight flight path, moths maintain an angle of fixed nature with respect to the moon. They are, however, severely disturbed by artificial light. Moths are seen to spiral towards artificial light and eventually latch onto the light [127], [128]. In the MFO algorithm, there exists a certain number of moths, and a certain number of flames. The population of moths and flames can be represented as shown in [127], with the fitness of the moths and flames represented as a matrix also shown in [127]. The initial random positions of the moths are given by [129]:

(30) $$m_{i,j}={lb}_{j}+rand({ub}_{j}-{lb}_{j})$$

Where $m_{i,j}$ is the position of the $i^{th}$ moth in the $j^{th}$ dimension, *rand* is a randomized number which lies in the range [−1,1] and $ub_{j}$ and $lb_{j}$ are the upper and lower limits of the boundary range respectively. The position of each moth is updated according to [129]:

(31) $$m_{i,j}(t+1)=D_{i,j}\times e^{ba}\times \cos(2\pi a)+f_{i,j}\;\text{if }i\leq F_{N}$$

(32) $$m_{i,j}(t+1)=D_{i,j}\times e^{ba}\times \cos(2\pi a)+f_{b}\;\text{if }i>F_{N}$$

Where $m_{i,j}(t+1)$ is the updated position of the $i^{th}$ moth in the $j^{th}$ dimension, $f_{i,j}$ is the current position of the $i^{th}$ flame in the $j^{th}$ dimension, $f_{b}$ is the position of the best flame, *a* is a randomized number in the range [−1,1], *b* is the shape constant and $D_{i,j}$ is the absolute value of the difference between $f_{i,j}$ and $m_{i,j}(t)$. $m_{i,j}(t)$ is the current position of the $i^{th}$ moth in the $j^{th}$ dimension.

(33) $$F_{N}=roundup(N-\frac{t\times (N-1)}{t_{max}})$$

Where *N* is the maximum number of moths, *t* is the current iteration and $t_{max}$ is the maximum number of iterations.

**Figure 17:**
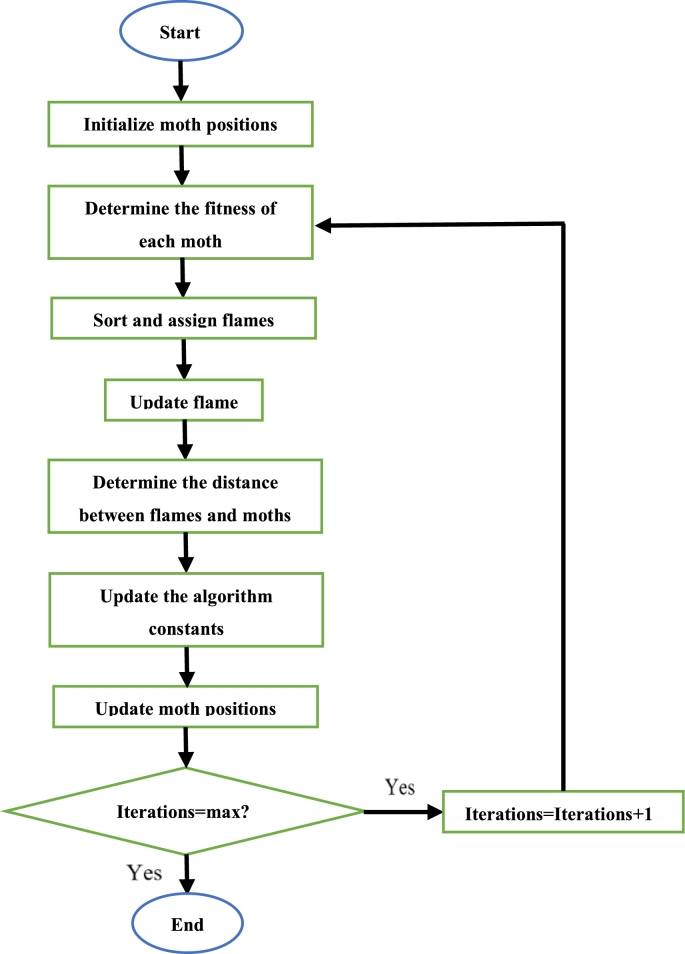
MFO algorithm flowchart [130].

Initially, the required parameters are defined. Then, random positions are assigned to all the moths according to (30). The fitness of each moth is then computed. After this, the positions of all the moths are updated according to (31) and (32). The fitness of each moth is then calculated again. If the current cost value is superior to the previous fitness value, the moth takes on the new fitness value (hence position). This continues until all iterations have been completed, each time updating the position of each moth of the fitness of the updated position is superior to the current best fitness of the moth. The value of $F_{N}$ is decremented at the start of each iteration. Once all iterations have been executed, the moth with the best fitness value is said to be the optimal solution [129]. The steps to execute the MFO algorithm can be seen in Fig. 17 [130].

The MFO has the merit of having a robust selection capability [131], with its demerits being a slow convergence rate and being easily entrapped in the local optima [128], [131], [132]. The authors in [131] attempted to overcome the demerit of being easily trapped in local minima by modifying the update formulas of both the moth and flames. It does so in three ways; the use of a levy flight equation, the use of a weighting factor and via a descending curvilinear strategy. The levy flight equation is applied to the entire of (31) and (32). The weighting factor is applied the last term in these two equations. The updating of the number of flames is done via a curvilinear fashion, using an equation that relates to the total number of flames and current and maximum iteration numbers. The proposed method was applied to the subthreshold image segmentation problem and compared to various swarm-based MOT, including the conventional MFO. The results show that on average, the proposed algorithm was the most superior in terms of global search capability. Of the 96 tests done, the proposed algorithm was only inferior to the conventional MFO in seven tests. In terms of convergence, the proposed algorithm exhibited dominance after a mere 10 iterations. A convergence factor that is reduced linearly from −1 to −2 was introduced in [128]. This enhances the global searching capability of the algorithm while also increasing the convergence rate. The convergence factor is a function of the current and maximum iteration number.

To further enhance the global searching capability, the flame number update equation is modified and is a function of the total number of flames, as well as the current and maximum iteration numbers. The proposed algorithm is employed to locate the optimal placement and sizing of distributed generator units, is applied to the IEEE-69 bus radial distribution test system and compared to various swarm-based MOT which includes the conventional MFO. The proposed algorithm offers a superior performance with regards to the sizing and optimal placement of distributed generators. In terms of the convergence rate, the proposed algorithm required a maximum of 30 iteration before superiority was established, validating a strong speed of convergence. The scholars in [133] proposed a novel swarm moth flame optimization algorithm for the tuning of four PI controllers which are responsible for controlling a DFIG. When compared to other MOT, the proposed method was shown to improve maximum power point tracking and enhanced fault ride through capabilities.

### 4.10 Sailfish optimization

Based on the group hunting behavior of the sailfish, sailfish optimization (SFO) is a model of the interaction between the sailfish and their prey, the sardine. Being the fastest animal underwater, the sailfish can reach a speed of 100 km/h. They hunt sardines by driving them to the surface of the water. Their immense speed makes it difficult for sardines to escape, but sardines do have good maneuverability. A sailfish uses its rostrum to slash and injure a sardine, or directly touch it and destabilize it. In the sailfish algorithm, both the sailfish and sardines are critical aspects to consider [134]. The positions of each sailfish and sardine can be represented as shown in [134], with the fitness values of the sailfish and sardines represented in a matrix also shown in [134]. It was previously mentioned that sailfish attack and subsequently injure sardines. This phenomenon should be incorporated into the sailfish algorithm to ensure that they have a substantial influence on the algorithm performance The elite sailfish and injured sardines are ones which have the best fitness values among their respective populations [134], [135]. The new position of the sailfish can be estimated using [134], [135]:

(34) $$x_{S}(t+1)=x_{SE}(t)-\lambda_{i}(rand\times (\frac{x_{SE}(t)-x_{Sal}(t)}{2})-x_{S}(t)$$

Where $x_{S}(t+1)$ is the updated position of the sailfish, $x_{S}(t)$ is the current position of the sailfish, $x_{SE}(t)$ is the current position of the elite sailfish, $x_{Sal}(t)$ is the current position of the injured sardine, rand is a randomized number which lies in the range [0,1], $\lambda_{i}$ is a function of a randomized number which lies in the range [0,1] as well as the prey density (PD). As the sailfish continues to hunt, the density of prey decreases. The PD can be calculated by utilizing the number of sardines and sailfish present. The number of sailfish is based on the initial sardine population. The initial sardine population is taken to be larger than the population of the sailfish [134], [135]. Initially, sardines have a high escape rate. However, due to the increase injuries and decrease in energy, the sardines are eventually caught. This can be mimicked by updating the position of the sardines using [134], [135]:

(35) $$x_{Sa}(t+1)=rand\times (x_{SE}(t)-x_{Sa}(t)+AP)$$

Where rand is a randomized number in the range [0,1], $x_{Sa}(t)$ is the updated position of the sailfish, $x_{Sa}^{t}$ is the current position of the sailfish and AP is the attack power of the sailfish which is based on the use of two independent constants, as well as the current iteration number. To allow convergence, the number of sardines and number of variables is reduced after each iteration [134], [135]. To increase its chances of catching more prey, the sailfish takes the position of the sardine that it has caught. This can be seen as [134], [135]:

(36) $$x_{S}=x_{Sa}\;\text{if }f(x_{Sa})<f(x_{S})$$

Where *f* represents the value of the fitness function. Once this occurs, the sardine that was caught is eliminated from the population [135].

**Figure 18:**
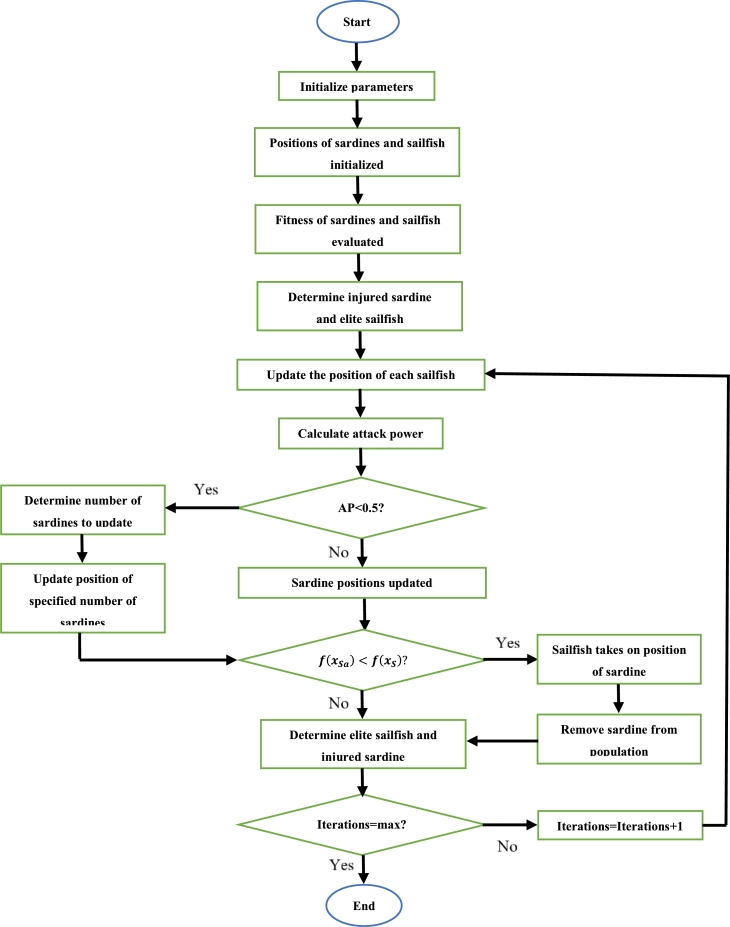
SFO algorithm flowchart [136].

Initially, the required parameters are defined. The fitness of each sailfish and sardine is calculated and the best values are noted. The position of each sailfish is then updated according to (34). The attack power is then calculated. If this value is less than 0.5, the values of *α* and *β* are estimated and used to update the position of a certain number of sardines by using (35). Else, the positions of all the sardines are updated. The fitness of each sailfish is then calculated once more. If this value is superior to the current best fitness value of that specific sailfish, the sailfish updates its position. Else it remains in its previous best position. The same rule applies to the sardines. Furthermore, if the fitness value of a sardine is superior to that of a sailfish, the sailfish takes on the fitness value (hence position) of the sardine. This sardine is then eliminated from the system. This continues until all iterations have been completed. Once this is so, the sailfish with the best fitness is said to be at the best solution [134], [135]. The steps to execute the SFO algorithm can be seen in Fig. 18 [136].

The conventional SFO has the merits of a fast convergence rate and being not easily trapped in the local optima [134]. To the authors best knowledge, there has been no established demerits of the SFO algorithm. This does not mean that none exist, but rather points to the lack of application of the algorithm.

### 4.11 Cuckoo search algorithm

Proposed by Yang and deb, the cuckoo search algorithm (CuSA) is based on the reproductive behavior of the cuckoo bird. Cuckoos are parasitic birds, which lay their eggs in other bird's nests. To prevent their eggs from being thrown out by the host bird, the female cuckoo lays eggs which imitate the eggs of the host in terms of factors such as shape and color. Cuckoo eggs usually hatch prior to the eggs of the host, and when this happens the cuckoo chick kicks out the host eggs to increase their share of food [137], [138]. There are three rules that govern the cuckoo search algorithm [137]. The first rule is that each cuckoo bird only lays one egg and places it in a random nest. The second rule states that the best nest (which contains the best quality eggs) has the best chance of being carried over to the next generation. The best nest is that in which the host eggs look very similar to the cuckoo eggs. The third and final rule states that the number of nests is unchangeable. Furthermore, there exists the probability of the host bird finding the cuckoo egg and either abandoning it or throwing it out. Either way, the egg would not survive. Using Levy flight, the random movement of the cuckoo bird to find a nest can be expressed as [138], [139]:

(37) $$x_{i}(t+1)=x_{i}(t)+\alpha \sigma Levy(\lambda )$$

Where $x_{i}(t)$ is the current position of the cuckoo, $x_{i}(t+1)$ is the next position of the cuckoo, *α* is a randomized number which is usually 1, *σ* is element wise multiplication, *λ* is a randomized value between 1 and 3 and Levy(λ) is a function of the current iteration number and *λ*. The following is used to update the position of the nest to allow the cuckoo to lay another egg [139]:

(38) $$x_{i}^{\prime }=\left\{ \begin{array}{cc} x_{i}+r(x_{r1}-x_{r2}),\; & \mathrm{rand}[0,1]>\mathrm{Pa}\\ x_{i},\; & \text{otherwise} \end{array}\right.$$

Where $x_{i}^{\prime }$ is the updated position of the nest, *r* and rand [0,1] are randomized numbers in the range [0,1], *r*1 and *r*2 are different randomized integers with a maximum to that equal to the number of cuckoos, Pa is the probability of the host bird identifying and discarding the cuckoo egg and is a randomized number in the range [0,1].

**Figure 19:**
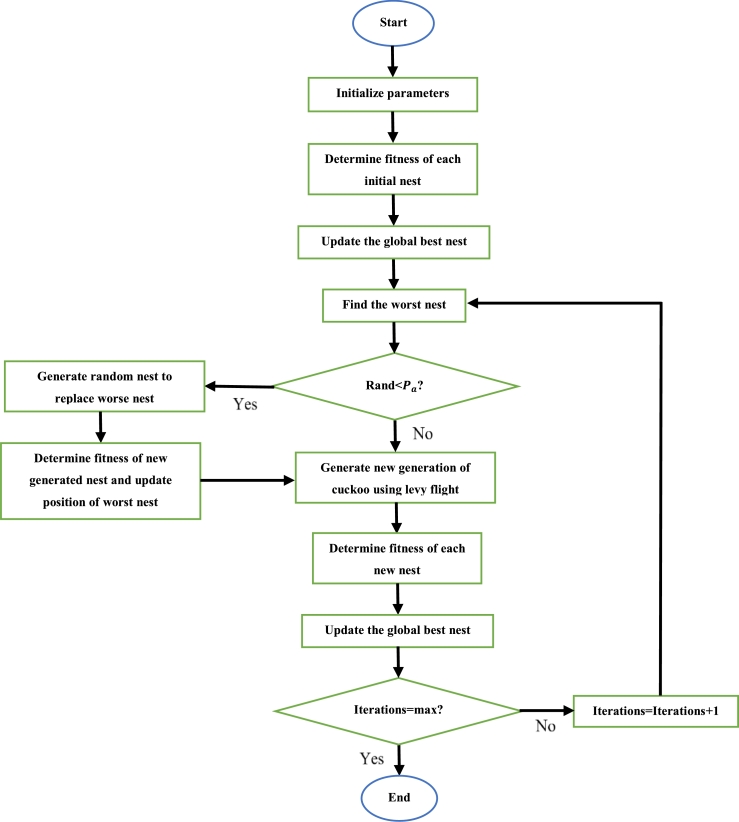
CuSA flowchart [140].

Initially, the required parameters are defined. The fitness of each nest is then calculated. Then, the position of the cuckoo is updated using (37). A nest is chosen at random, and its fitness value is compared to the fitness value of the cuckoo. If the fitness value of the nest is superior to that of the cuckoo, the cuckoo takes on the fitness (and hence position) of the nest. A fraction of nests with the poorest fitness values are eliminated and is replaced according to (38). This continues until all iterations have been completed. Each time, the cuckoo updates its fitness values (and hence position) if the fitness of the nest that it is compared to is superior to its own fitness. Once all iterations have been implemented, the position of the best cuckoo is said to be the optimal solution [139]. The steps to execute the CuSA algorithm can be seen in Fig. 19 [140].

The conventional CuSA has the merit of requiring knowledge of just a few parameters [141] but suffers the demerits of a stagnant rate of convergence [142] and being easily trapped in the local optima [141], [142]. The authors in [141] incorporated the method of differential evolution in (37). In this proposed modification, the position update of a particular cuckoo is based on the position of three random cuckoos. The proposed algorithm was implemented on monopulse antenna problems in 20-element arrays, 40-element arrays, and a fixed number of subarrays. The proposed algorithm was compared to various MOT, including the conventional CuSA. In the 20-element array, five subarrays were tested. It was noticed that the proposed algorithm produced the best global search capability in all five subarrays. In the 40-element arrays, 10 subarrays were tested. It was observed that the proposed algorithm produced a superior global search capability in all 10 subarrays. The same is true for the five subarrays tested for the fixed number of subarrays. This clearly confirms the superiority of the proposed algorithm.

The scholars in [142] made use of a coefficient function to change the step size. The proposed algorithm also makes use of a logistic map of each dimension to initialize the location of the host nest and update the position of the host nest beyond the boundary. The proposed algorithm was tested on fifteen benchmark functions and compared to the conventional CuSA. It was observed that the proposed algorithm produced a superior global search capability in all the tested functions. With regards to the convergence rate, the proposed algorithm proved to be superior in all fifteen functions, exhibiting dominancy upon the completion of a maximum of 20% of the total number of iterations.

CuSA was applied to both a PI controller and FOPID controller to control a DFIG in [143]. The method applied this control to the pitch controller, RSC and GSC. The method also made use of all the common performance indices (ITAE, IAE, ISE, and ITAE) and combined all of these to form the objective function to be used. The objective function was a sum of these common performance indices, with each index multiplied by a weighting factor. The cumulative sum of the weighting factors is one, and the weighting factors were determined using the (CuSA). For the pitch controller and RSC controllers, the PI controller produced the best results and for the GSC controller, the FOPID controller produced the best results. However, results were only given in terms of errors derived from the use of the different performance indices. Very little graphical results were provided, and no steady state and dynamic response comparisons were provided. Furthermore, there was no comparison with other MOT. This made it difficult to validate the results provided.

### 4.12 Firefly algorithm

Developed in 2007 by Yang, the firefly algorithm (FA) is based on the behavior and patterns of flashing of fireflies [106]. This optimization algorithm uses four rules [144], [145]. The first rule states that the less bright fireflies are attracted to brighter fireflies and this attraction occurs without any regard for gender. The second rule is that the brighter a firefly appears to be, the more attractive it seems. The third rule says that the further away firefly *a* is from firefly *b*, the less attractive it appears. In the fourth and final rule, the brightest firefly is the only firefly that moves randomly. As mentioned, the light intensity of a firefly relative to another firefly is dependent on the distance between the two. This can be expressed as [144], [145], [146]:

(39) $$L=L_{0}e^{-{\delta y}^{2}}$$

Where $L_{0}$ is the maximum light intensity, *L* is the light intensity of firefly *b* as seen by firefly *a*, *y* is the distance between the two fireflies and *δ* Is the light absorption which is dependent of the medium in which the firefly exists. It is usually between 0.1 and 10. The attractiveness, *B*, of a firefly is dependent on the light intensity seen by the other firefly and is estimated in the same manner as the light intensity [144], [145], [146]. The movement of firefly *a* to firefly *b* can be expressed as [144], [145], [146], [147]:

(40) $$x_{a}(t+1)=x_{a,k}+B_{0}e^{-{\delta y}_{a,b}^{2}}(x_{b,k}-x_{a,k})+\alpha (rand-0.5)$$

Where rand and *α* are randomized numbers in the range [0,1], $B_{0}$ is the maximum attractiveness, $x_{a}(t+1)$ is the updated position of firefly *a*, $x_{a,k}$ and $x_{b,k}$ are the current positions of fireflies *a* and *b* respectively in dimension *k*, $y_{a,b}$ is the distance between fireflies *a* and *b*.

**Figure 20:**
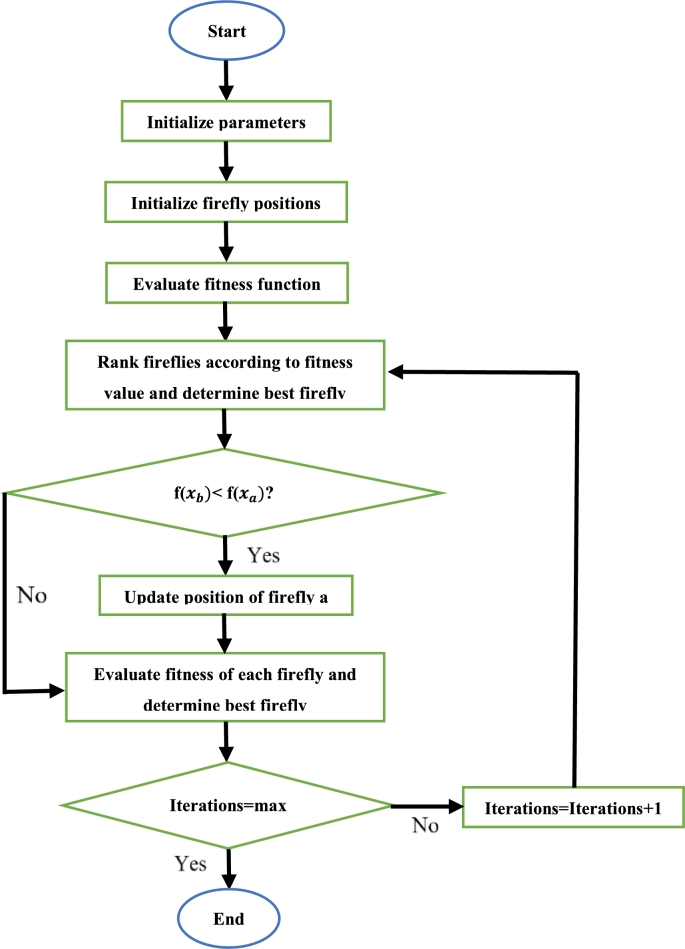
FA flowchart [148].

Initially, the required parameters are defined. Then, each firefly is assigned a random position. The fitness of each firefly is then computed. Thereafter, the fitness value of each firefly *a* is compared to the fitness value of a randomly chosen firefly *b*. If the fitness of *b* is superior to *a*, then the position of *a* is updated using (40). Once this process is complete, the fitness value of the possibly updated positions of each firefly is computed. If this fitness value is superior to the previous fitness value of that specific firefly, that firefly updates its fitness (and hence position) to the better value. This continues until all iterations have been completed. Once this is so, the firefly with the best fitness is said to be at the best position [144], [145], [146], [147]. This is seen in Fig. 20 [148].

The conventional FA suffers the demerits of being easily trapped in the local optima [146], [149] and a slow convergence rate [146], [150]. The authors in [149] added the concept of velocity to (15.2) to both improve the global search capabilities and enhance the algorithm rate of convergence. The velocity factor utilizes the concept of randomness, acceleration coefficients and the position of the $i^{th}$ firefly. The proposed algorithm was employed to the design of a digital infinite impulse response filter and compared to the conventional FA. The proposed algorithm outperformed the conventional algorithm; it produced a lower mean square error and a superior rate of convergence.

Considering a second order system with a second order filter, the proposed algorithm exhibited a minimum mean square error dominance of 6.92%, with a maximum superiority of 26.77%. This corresponded to 0.61% and 1.36% respectively for a second order system with a first order filter. The scholars in [146] proposed various modifications to the conventional FA. The randomization factor, absorption coefficient and initial attractiveness are all constants. This decreases the convergence speed of the algorithm. The first improvement is to change these values from constants to dynamic variables. The modified absorption coefficient is a function of the current iteration number and a randomized number in the range [0,1]. The randomization factor and initial attractiveness are both functions of the current and maximum iteration numbers. In the second improvement, the influence of the global best is considered in the position update equation. If the intensity of firefly *a* is less than *b*, then this term is added to (40). If the intensity of firefly *a* is greater than *b*, the term replaces term 2 in (40). In the third improvement, the search space is updated after each iteration. This is done with respect to the global best firefly and “squeezes” the fireflies to the global optimum. The proposed algorithm was applied to a highly nonlinear and multi model dispatch problem and compared to the conventional FA. Two cost functions were tested. For each cost function, various populations of fireflies were tested. These are 5, 15, 35 and 50 fireflies. For case 1, it was observed that the proposed algorithm produced a superior global search capability for all four firefly populations. For case two, the proposed algorithm is inferior to the conventional FA for the 5 firefly population. In the scenarios whereby the proposed algorithm yielded superior results, this occurred before completion of 5 iterations. Therefore, it can be concluded that the proposed algorithm exhibited a superior rate of convergence

Considering the application of the FA to the control of the DFIG, the authors in [151] made use of a second order lead lag power oscillation damper (POD). The parameters of the POD are optimized by the FA. The aim of the algorithm is to stabilize inter area oscillations in interconnected power systems by means of a POD equipped with a DFIG. The proposed robust DFIG-POD was compared to the conventional DFIG-POD. The proposed method showed a superior damping performance with regards to heavy power flows, fault location, severe faults, and varying patterns of wind. However, no comparison or evaluation of the optimization technique was provided. Reference [152] makes use of a hybrid PSO and FA for the regulation of a multi area power system's frequency. The power system contains DFIG's. Two different controllers were tested: PID controller and a cascaded PD-PI controller. To analyze the dynamic performance of the system, a 1% load disturbance was injected into the system. The fitness function used was the ITAE. The hybrid PSO/FA was applied to both the PID controller and PD-PI controllers. The proposed method modifies the conventional PSO velocity equation by replacing the acceleration constants with FA parameters. The results confirm that the latter controller surpasses the PID controller in all aspects. However, the proposed metaheuristic optimization technique was not compared to other control techniques, hence its superiority could not be validated.

### 4.13 Shuffled frog leaping algorithm

Introduced by Eusuff and Lansely, the shuffled frog leaping algorithm (SFLA) is inspired the hunting strategy of frogs. The SFLA is known to have favorable performances of both GA and PSO. In SFLA, a group of frogs are divided into groups, each group known as a memeplex. Each memeplex performs a local search. Via the process of memetic evolution, each frog evolves based on the ideas of other frogs. After a predetermined number of memetic steps has occurred, information is shuffled among the different memeplexes [153], [154]. The SFLA is divided into 4 steps, namely initialization, partition, updating and shuffling:

*Initialization: Considering a frog to be a solution to the problem, an initial population of frogs is randomly generated. The*$i^{th}$*frog's position is denoted as:*
  (41) $$X_{i}=(x_{\mathrm{i1}},x_{\mathrm{i2}}\ldots x_{\mathrm{id}})$$
  Where *d* is the search space dimension (number of variables to be optimized)
*Partition: Each frog is put into a memeplex, the total number of memeplexes being m. This sorting is done in descending order, based on the fitness value of each frog. Once each memeplex contains one frog, the frogs are again placed into a memeplex. Each memeplex would therefore have n frogs*[153], [154].
*Updating: In each memeplex, the position of the worst frog (with the poorest cost function) is updated according to*[153], [154]:
  (42) $$D=\mathrm{rand}\times (X_{b}-X_{w})$$
  (43) $$X_{w}^{n}=X_{w}+D$$
  (44) $$-D_{\max}\leq D\leq D_{\max}$$
  *Where*$X_{b}$*is the position of the best frog in each memeplex*, $X_{w}$*is the position of the worst frog in each memeplex*, $X_{w}^{n}$*is the updated position of the worse frog*, $D_{max}$*is the maximum specified value of D. If this process fails to produce a superior solution, then*$X_{g}$, *which is known as the global best position is used in place of*$X_{b}$*and the process is repeated. If this still fails to produce a better solution, a random solution is generated in place of the position of the worst frog*[155].
*Shuffling: After a certain number of improvement processes, all the frogs in the population are mixed and the process of partitioning and updating is repeated. This is done until the termination criteria is met*[154].

**Figure 21:**
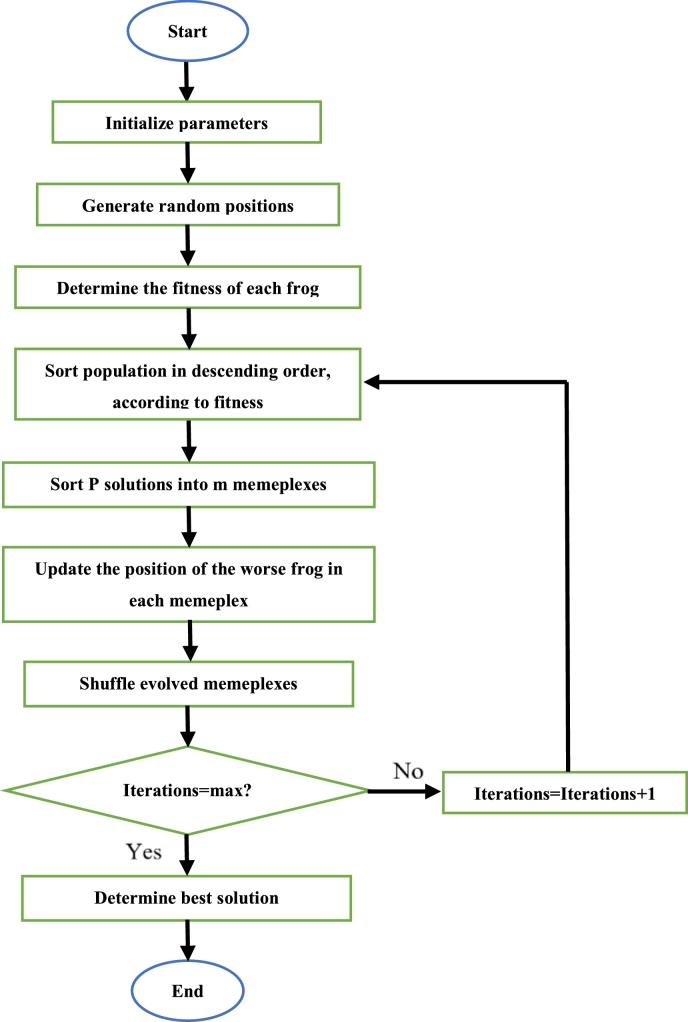
SFLA flowchart [156].

Initially, the required parameters are defined. Each frog is then given a random position. Afterwards, the fitness of each frog is computed, and the frogs are arranged in order from best to worst fitness. The frogs are then separated into memeplexes. The worst frog (with the poorest cost value) in each memeplex is updated using (43). If this fails to produce a better solution, the same equations are used again, this time replacing the best frog in each memeplex with the global best frog. If this also fails to produce a better solution, that frog is discarded and a new frog is placed in a random position. This continues until all iterations have been completed. Once this is so, the frogs are shuffled and sorted into memeplexes once again. This process is continued until the iterations are complete. Once this is so, the frog with the best fitness value is said to be at the optimal position [153], [154], [155]. The steps to execute the SFLA can be seen in Fig. 21 [156].

The conventional SFLA has the merit of a fast convergence speed [157]. However, it suffers the demerits of premature convergence to the local optima [157], [158] as well as random jumps which leads to blind searches [159]. The authors in [157] proposed a SFLA which introduces the application of a weighting factor based on chaos memory and an absolute balance group strategy. The chaos memory weighting factor aimed at improving the local and global search capabilities of the algorithm. This weighting factor is applied to the formula in (42) and hence used to update the worst frog's position. The weighting factor is a function of the position of the best and worst frogs, and a randomized number in the range [0,1]. The absolute balance group strategy aims to avoid premature convergence to local optima by modifying the strategy of sorting the frogs into memeplexes. It does so by randomly sorting the frogs into memeplexes, instead of using the fitness function value of each frog. The proposed algorithm was tested using the K nearest neighbor method and compared to evolutionary and swam-based MOT, including the conventional SFLA. The results showed that the application of both the chaos memory weighting factor and absolute balance group strategy produced superior global optimization performances. The proposed algorithm was applied to nine functions and compared to various MOT, including the original SFLA. It was observed that the proposed algorithm produced the most accurate result in 7 of the 9 cases. Considering stability, the proposed algorithms displayed a 66% success rate. However, it was observed that as the number of subsets (dimensions) increased, the performance of the proposed algorithm declined by a large extent. In many cases, the proposed algorithm was seen to be inferior to the other compared techniques. The converge curve, which is a critical piece of information when evaluating algorithms, was missing. This creates doubt in the confidence of the proposed scheme.

The scholars in [159] present a modified SFLA. In this method, in each memeplex, the worst frog's position is updated using the position of the local best and local worst frogs as well and a randomized number which lies in the range [0,1]. If the fitness value of the updated position is worse than the previous position, the position of the worse frog is again updated, this time using the position of the global best instead of the local best (best in each memeplex). If there is still no improvement, the method makes use of cloning of frogs. Two types of cloning can occur. In the first type, the frogs with the best positions are cloned. In the second method, a frog is cloned at random. The position of the new frog as a result of the cloned frog is a function of the position of the cloned and worst frog and a randomized number in the range [0,1]. If this method also fails to produce a better solution, the frog in question is discarded from the memeplex and replaced with a randomized frog. The proposed method is tested using the Markov chain theory and compared to various MOT, including the conventional SFLA. It was observed that in all the tests performed, the proposed algorithm produced the best global search capability. From the results provided, it is difficult to accurately compare the convergence rate of the different techniques. However, the author claims that the proposed algorithms produce a superior convergence rate.

A new method to update the position of the worst frog is introduced in [158]. The proposed method updates the position of the worst frog using the position of the frog in the center of each memeplex, as well as a randomized number in the range [0,1]. This simple proposed method was employed to the optimization of the path of a robot under both static and dynamic environments and fared against the conventional SFLA. Considering the static environment, the proposed algorithm yielded a search accuracy superiority of 25.28%. The proposed algorithm also achieved the task 14.18% quicker. The superiority of the proposed algorithm for the dynamic environment was 36.85% and 34.3% respectively for the search accuracy and task completion time.

### 4.14 Antlion optimization

Antlion optimization (ALO) is an algorithm inspired by the foraging behavior of antlion larvae. Its formation lies on the basis of the relationship between the antlion and their prey, the ant. Antlions dig a hole of conical shape in the ground and hide at the pit to catch prey. Hungrier ant lions dig bigger holes which improves their chances of catching prey. The ants slide down the surface of the hole, at which point the antlion consumes it [160], [161]. Ants move randomly. Their movement is affected by the holes dug by the ant lions. Ant lions with poorer fitness functions dig bigger holes. In each iteration, every ant can be trapped by an antlion. To simulate ants sliding down the surface of the holes, the range of the random walks is adaptively decreased. If the value of the fitness function of an ant is superior to that of the antlion, it means that the ant is caught by the antlion. The, the antlion repositions itself to the caught prey's position. This increases its chances of catching another prey [160], [161]. Mathematically, the random walk of the prey (ants) can be described as [161]:

(45) $$x_{t}=[0,cs(2r(t_{1})-1)\ldots ,cs(2r(t_{n})-1)]$$

Where *cs* is the cumulative sum, *n* is the maximum number of iterations, *t* is the iteration step and *r* is a randomized number in the range [0,1]. Normalizing this in a search space (to allow for consideration of boundaries), the following is obtained [160], [161]:

(46) $$X_{i}^{t}=\frac{\left(X_{i}^{t}-a_{i})\times (d_{i}^{t}-c_{i}^{t}\right)}{b_{i}-a_{i}}+c_{i}^{t}$$

Where $X_{i}^{t}$ is the position of the $i^{th}$ ant at iteration *t*, $b_{i}$ and $a_{i}$ represent the maximum and minimum values of the random walk for the $i^{th}$ ant and $d_{i}^{t}$ and $c_{i}^{t}$ represent the maximum and minimum values of the $i^{th}$ ant at iteration *t*. To ensure that the position of each ant always stays within the boundary, (50) should be computed at each iteration. Mathematically representing the effect of the holes dig by antlions on the random walks of ants can be seen as [161]:

(47) $$c_{i}^{t}={Antlion}_{j}^{t}+c_{t}$$

(48) $$d_{i}^{t}={Antlion}_{j}^{t}+d_{t}$$

Where $Antlion_{j}^{t}$ is the position of the $j^{th}$ antlion at iteration *t*. The antlion is selected by using the roulette wheel method, allowing the hungrier antlions a greater probability of catching an ant.

It was observed that when an antlion notices an ant in its trap and trying to escape, it throws sand at the ant to make the ant slide further down. This is modelled by adaptively decreasing the search space and is shown as [161]:

(49) $$c_{t}=\frac{c_{t}}{I}$$

(50) $$d_{t}=\frac{d_{t}}{I}$$

Where *I* is a ratio of the current to maximum iteration numbers. It was mentioned that if the fitness of the ant is superior to that of the antlion, the antlion then updates its position to that of the ant. Based on the premise that if the ant fitness it greater than that of the antlion then the ant is said to be captured, this means that updating of the antlions position occurs after the antlion has consumed the prey.

This is shown as [161]:

(51) $${Antlion}_{j}^{t}={Ant}_{i}^{t}\;\text{if }f({Ant}_{i}^{t})>f({Antlion}_{j}^{t})$$

Where $Ant_{i}^{t}$ represents the position of the *i*th ant at iteration *t* and *f* is the value of the fitness function. Note that (55) is based on fitness function maximization. To ensure that the fittest antlion affects the outcome of the process, the ant's position is determined based on the random walk around a roulette wheel selected antlion, as well as their walk around the fittest (most elite) antlion. This can be expressed as [161]:

(52) $${Ant}_{i}^{t}=\frac{R_{A}^{t}+R_{B}^{t}}{2}$$

Where $R_{A}^{t}$ represents the walk of random nature around the antlion (selected via the roulette wheel) at iteration *t* and $R_{B}^{t}$ is the random walk around the fittest antlion at iteration *t*.

**Figure 22:**
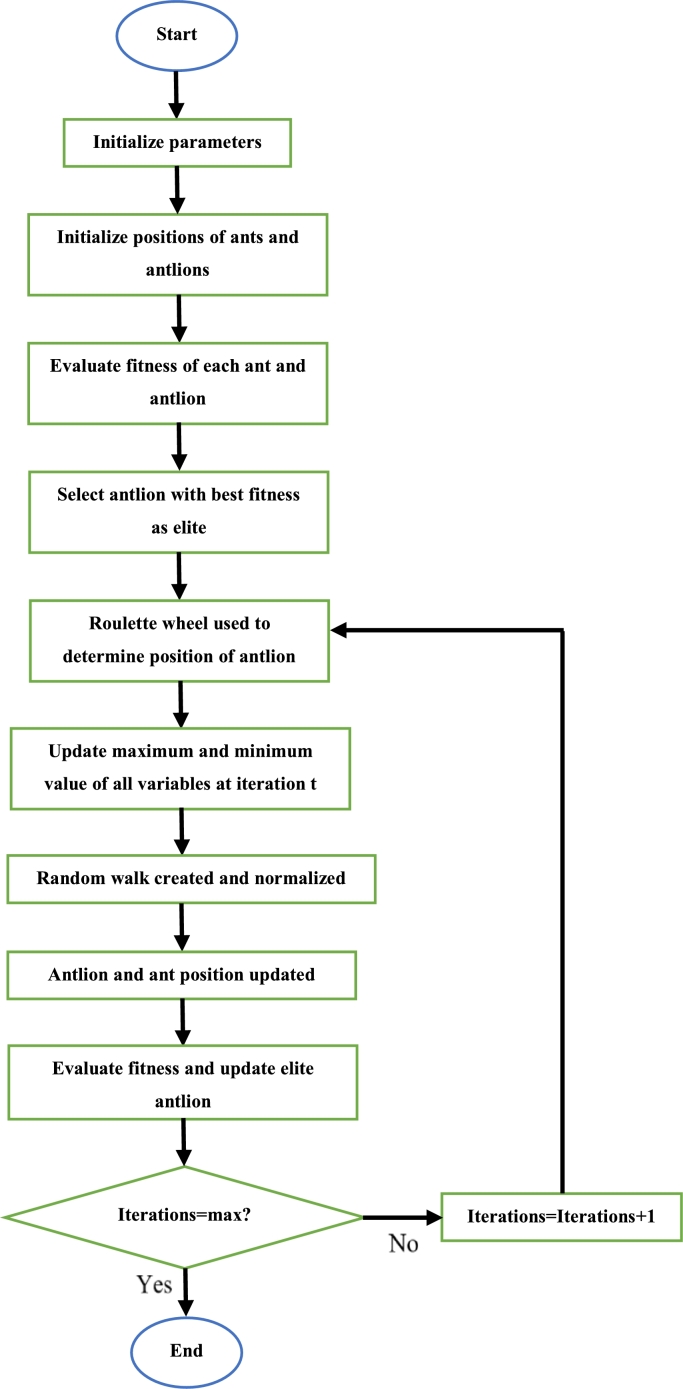
ALO algorithm flowchart [162].

Initially, the specific parameters are defined. Then, each ant and antlion are given randomized positions. The fitness of each antlion and ant is computed and the antlion with the best fitness value is noted as the elite antlion. Thereafter, the position of each ant is updated using (46) and (52). The fitness value of each ant is then computed, and if this fitness value is superior to the previous fitness value, then the ant takes on this new fitness value (hence position). For each ant, an antlion is chosen via the roulette wheel. If the fitness value of the ant is superior to that of the antlion, the antlion takes on the fitness value (hence position) of the ant. The elite antlion is then updated. This continues until all iterations have been completed. Once this is so, the elite antlion (with the finest fitness value) is considered to be the best solution [160]. The steps to execute the ALO algorithm can be seen in Fig. 22 [162].

The conventional ALO has the demerit of being easily trapped in local optima [163]. The authors in [163] proposed a spiral complex searching pattern to overcome this demerit. In total, eight spiral paths were applied. These are the Rose Logarithmic, Inverse and Archimedes spiral curves, as well as the Epitrochoid, Hypotrochoid, Cycloid, and Spiral based overshoot parameter setting. The proposed algorithms were applied to various unimodal, multimodal, and fixed dimension benchmarks functions and compared to the conventional ALO. Considering global search capability, superior performance for the different functions was seen to be scattered across the various proposed algorithm. It is vital to note though, that for each case, at least one proposed spiral complex searching pattern proved to be superior to the conventional ALO. Majority of the best results were obtained by the Spiral based overshoot parameter setting. It was also noticed that on average, the Spiral based overshoot parameter setting yielded the strongest convergence rate. The method proposed in [164] aims to improve the algorithm global search ability by proposing a modified ant position update equation. This considers the pheromones left behind by other ants. The proposed algorithm was applied to a bearing fault identification which is centered on multi-layer extreme learning machine (MELM). The proposed algorithm was employed to the optimization of the MELM. However, no comparison between the proposed algorithm and other MOT is provided. This means that the effectiveness of the proposed method to the said application cannot be verified.

Considering the application of ALO to the control of the DFIG, the authors in [165] make use of ALO to obtain the fractional order PI (ALO-FOPI) controller gains. The method makes use of stator flux-oriented control and considers both the RSC and GSC control. Apart from the ALO-FOPI control algorithm, two other control algorithms are tested. The first is a PI controller with an ANFIS controlled added. The required gains are optimized using the Cuckoo search algorithm (CuSA-ANFIS). In the second method, a hybrid CuSA and ALO algorithm was utilized to optimize the parameters of a fractional order PI controller (CA-FOPI). The proposed algorithms were tested at two wind speeds, names 15 m/s and 10 m/s. At both 10 m/s and 15 m/s, it was observed that ALO-FOPI and CA-FOPI produces an identical and superior stator reactive power percentage overshoot than Cu-ANFIS. For both wind speeds, all three control algorithms responded identically with regards to the dc link voltage regulation. The results presented for the stator active power were unclear hence could not be analyzed. Furthermore, to clearly compare ALO algorithm and CuSA, it would have been profitable to implement a PI and ANFIS controller which is optimized using ALO.

## 5 Summary and discussion of techniques reviewed

Table 1 summarizes the swarm-based MOT that were discussed in this paper. The summary state the merits, demerits, and application of each technique to the control of the DFIG. The development of PSO has been an excellent advancement in science. Since its inception, PSO has been used countless times to solve optimization problems. It has also paved the way for other swarm-based MOT to arise. However, theory upon which PSO stands is basic. Practically, flocks of birds and schools of fish display highly complex and intelligent behavior, which has not been incorporated into the algorithm. PSO is one of the few swarm-based MOT which displays fast convergence, hence modification and incorporation of intelligent behavior of fish and birds may result in PSO once again being a superior optimization technique. A similar observation holds true for GWO. The idea of utilizing the ranking of wolves within a pack was innovative, but the equations of the optimization tool are very basic. Wolves display exceptional survival tactics, and more careful observation of their behavior could result in alleviation of the algorithms disadvantages. The idea upon which CSA and CuSA stand are interesting, however the equations of these algorithms are not complex enough to simulate the relative behavior.

**Table 1:** Summary of reviewed swarm-based metaheuristic optimization techniques.

Swarm technique	Merits	Demerits	Application to the DFIG	Significant findings regarding application to DFIG
Particle swarm optimization	Fast convergence rate [66], [67]	• Poor accuracy • Easily trapped in local minima [66], [67], [68]	• Optimization of parameters of sliding mode controller [61] • Optimization of PI controller gains to analyze small signal stability [69] • Optimization of PID controller gains for standard control [71] • Optimization of FLWRBFN for stability enhancement of a DFIG based ocean energy conversion system • Optimization of dish Stirling system incorporating DFIG for maximum power point tracking enhancement and receiver temperature regulation	• Regarding PSO optimized sliding mode controller, emphasis was given to the efficacy of the proposed controller and not on the effectiveness of PSO with regards to parameters optimization • In the case of both small and large disturbances, PSO optimized PI controllers produce strong overshoots and damping rates • Considering standard control, PSO optimized PID controllers produce superior results in all aspects when fared against the supervisory control method • Considering PSO optimized FLWRBFN for stability enhancement, emphasis was given on the efficacy of the proposed controller and not on the effectiveness of PSO with regards to parameter optimization • Considering optimization of dish Stirling system incorporating DFIG, emphasis was given on the efficacy of the proposed controller, and not on the effectiveness of PSO with regards to parameter optimization
Bacteria foraging optimization	Not easily trapped in local minima [79]	Not yet established	• PI control gain optimization for standard control [48] • PI controller gain optimization for damping of low frequency oscillations [80]	Considering standard control, BFO optimized PI controllers produce superior results in all aspects when compared to PSO
Grey wolf optimization	Strong local search capability [82]	• Poor global search capability • Slow convergence at the latter part of the algorithm [81], [82], [83]	Optimization of FOPID controller gains for standard control [85]	When compared to PSO-PID and BFO-PID the GWO optimized FOPID controller produced a superior rise time, settling time and overshoot. However, it proved to be inferior to BFO-PID when considering disturbance rejecting capabilities.
Artificial bee colony	Strong global search capability [93]	Slow convergence rate [94], [95], [96]	Optimization of PI controller gains for standard control [98]	ABC optimized PI controllers produce superior overshoots to PSO and GWO optimized PI controllers, but an inferior dynamic response
Whale optimization algorithm	Strong global search capability [105]	• Poor accuracy • Slow convergence rate [106]	Not yet established	Not yet established
Crow search algorithm	• Flexible • Requires knowledge of only a few parameters	• Easily trapped in local minima • Slow convergence rate [110]	Optimization of PI controller gains for standard control [112]	CSA optimized PI controllers produced mixed results when fared against Genetic Algorithm and the supervisory control method. CSA proved to be an unsuitable method for PI controller tuning for standard control
Bat algorithm	Fast convergence rate [118]	• Poor accuracy • Easily trapped in local minima [114]	Optimization of parameters of sliding mode controller [123]	BA optimized sliding mode controller was superior to the conventional sliding mode controller and PI controller tuning with respect to rise time and settling time. However, there did exist a minor unbalance in the stator voltage waveforms
Squirrel search algorithm	Strong stability [124]	• Poor accuracy • Easily trapped in local minima [124]	Not yet established	Not yet established
Moth flame optimization	Robust selection capability [131]	• Easily entrapped in local optima • Stagnant convergence rate [128], [131], [132]	Optimization of PI controller gains for standard control [133]	When compared to various other MOT, MFO optimized PI controllers displayed enhanced maximum power point and fault ride through capabilities
Sailfish optimization algorithm	• Fast convergence rate • Not easily trapped in local minima [134]	Not yet established	Not yet established	Not yet established
Cuckoo search algorithm	Requires knowledge of only a few parameters [141]	• Slow convergence rate [142] • Easily trapped in local minima [141], [142]	• Optimization of PI controller gains for standard control • Optimization of FOPID controller gains for standard control [143]	Regarding both the PI and FOPID controllers optimized using CuSA, very little analysis was provided. Critical aspects such as rise time and settling time were not considered. Further, there was no comparison to optimization using other MOT
Firefly algorithm	Not yet established	• Slow convergence rate [146], [149] • Easily trapped in local minima [146], [150]	• Optimization of POD controller gains for stabilization of inter area oscillations [151] • Optimization of PI-PD controller gains for frequency regulation • Optimization of PID controller gains for frequency regulation [152]	• Considering FA optimized POD, emphasis was given on the proposed control structure, and not on the effectiveness of the optimization technique • Regarding FA optimized PI-PD and PID controllers, the PI-PD controller was superior in all aspects concerning frequency regulation.
Shuffled frog leaping algorithm	Fast convergence speed [157]		Not yet established	Not yet established
Antlion optimization algorithm	Not yet established		Optimization of FOPI controller gains for standard control	ALO optimized FOPI controller seems to be promising in terms of standard DFIG control.

ABC, WOA, BA and SSA are complex in structure, and account for many of the characteristics of the respective swarms. However, some key aspects are missing. For example, in a bee colony, the queen bee plays an important role in the colony. The effect of incorporating the behavior of the queen bee should be investigated. Likewise, it may be beneficial to consider the hunting strategies of other whales so as to broaden and enhance the capabilities of WOA. SSA considers only the foraging behavior of flying squirrels. This could be broadened to incorporate the behaviors of other types of squirrels. A similar suggestion is given to the BA, which is based on the behavior of the microbat. BA and SSA have thus far looked promising, but they have yet to be extensively tested. To validate their capabilities, rigorous testing and application is required. The same applies for ALO, SFLA, MFO, SFO and FA. Regarding ALO, the mathematical representation of the holes dug is simple. This type of representation should be investigated thoroughly so as to ensure strong simulation of the effect of these holes on ants. It is observed that of the techniques discussed, BFOA is by far the most complex. It is evident that this technique incorporates most, if not all, of the behavioral traits of the E. Coli bacterium. This can be attributed to the large amount of literature concerning this bacterium. BFOA seems to have a lot of undiscovered potential, which should be researched.

Various comparisons between conventional techniques are presented in literature. A comparison between ABC and PSO is presented in [166]. When tested on various unimodal and multimodal benchmark functions, it is observed that both algorithms display identical characteristics in the case of unimodal functions. For multimodal functions, ABC outperforms PSO. It is also observed that ABC is more sensitive to population and dimension sizes. This opens a wide area of research, as the effects of these parameters were not investigated in current literature. Another comparison is presented in [167], where PSO, FA, ABC, CSA and GWO are fared against each other. Considering unimodal functions, GWO was superior in 6 of 7 functions. GWO also produced good results in fixed dimensional multimodal functions. However, this is not the case for standard multimodal functions. CSA produced the worst average results among all the techniques, showing the strong need for improvement. A comparison between PSO and SFLA is provided in [168]. The results show that SFLA produced the overall best convergence rate and search accuracy. However, unlike PSO, it was found that SFLA is highly sensitive to user defined parameters, in particular the number of frogs and number of memeplexes. BA and BFO are fared against each other in [169]. When applied to a wide range of benchmark function, it was observed that BFO was superior in terms of accuracy. However, BA produced a faster convergence rate.

**Figure 23:**
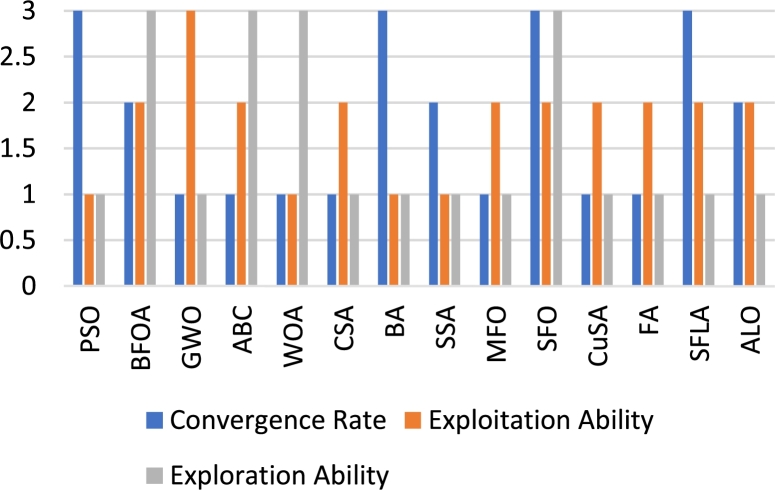
Comparison of discussed techniques in terms of convergence rate, exploitation, and exploration capabilities.

From Table 1, it is observed that many of the discussed swarm-based MOT suffer the demerit of being easily trapped in the local optima. The good news, however, is that various advancements have been made to correct this. It is also seen that the demerits of some MOT have not yet been established. This does not necessarily mean that none exist, but rather points to a lack of investigation into the operating capability of the algorithm. With regards to the application of swarm-based MOT to the control of the DFIG, it is observed that PSO is the most established in this aspect. Other techniques have been applied once or twice, but not comprehensively. However, the efficacy of PSO as an optimization tool For DFIG control is not well validated. A similar issue is observed with CuSA and FA. With regards to DFIG application, The BA and ALO show promise, but require much more rigorous testing to be validated. The CSA proved to be ineffective when applied to the DFIG, and modifications to this algorithm should be presented before considering reapplication of this algorithm to the DFIG. GWO and ABC have displayed positive results thus far, but it is evident that these techniques have room for enhancement. BFO and MFO show strong capabilities when applied to the DFIG. However, these techniques have not been extensively applied to the DFIG, hence their efficacy is yet to be validated. Lastly, it is seen that some swarm-based MOT are yet to be applied to the control of the DFIG. Examples of these are SFO, SSA and SFLA. Fig. 22 provides a visual representation of the convergence rate, exploitation and exploration capabilities of each of the algorithms discussed. In Fig. 23, a value of 1 represents a weak capability, 2 represents an average capability, a 3 represents a strong capability. From Fig. 23, it is evident that various swarm-based MOT have a weak capability when it comes to exploration, and majority of the algorithms have an average capability when considering exploitation. Considering an equal weighting of all three factors, it is observed that the SFO provides the best overall response. Therefore, its lack of application to the control of the DFIG is an interesting and possibly promising area of research.

Table 2 provides a method for choice of algorithm for specific applications to the DFIG. From this Table, it is observed that should one want to attempt to optimize PI controllers for standard control of the DFIG, various algorithms may be used. In the situation where optimization of FOPID controller gains for standard control is required, the CuSA and GWO seem the best choice. For optimization of parameters of sliding mode controllers, PSO and BA should be the algorithms utilized. It is important to note, though, that the application of swarm-based MOT to the control of the DFIG has not been achieved extensively. This means that, for the objectives defined in Table 2, various other swarm-based MOT (in addition to the ones presented) have the potential to yield desired results.

**Table 2:** Choice of algorithms for specific application to DFIG.

Objective	Suggested technique/s
PI control gain optimization for standard control	PSO, ABC, CuSA, MFO
PI controller gain optimization for damping of low frequency oscillations	PSO
Optimization of FOPI controller gains for standard control	ALO
Optimization of FOPID controller gains for standard control	CuSA, GWO
Optimization of PI controller gains to analyze small signal stability	PSO
Optimization of PID controller gains for standard control	PSO
Optimization of parameters of sliding mode controller	PSO, BA
Optimization of POD controller gains for stabilization of inter area oscillations	FA
Optimization of PID controller gains for frequency regulation	FA

## 6 Simulation-based analysis of common swarm-based MOT

In this section, a simulation-based analysis of the results of various well-known techniques is carried out. These techniques are PSO, ABC, and WOA. These algorithms are applied to three benchmark functions, at three dimension magnitudes. The information regarding the test functions can be found in Appendix A. To allow for a fair comparison, the number of search agents and particles were kept uniform across all three algorithms. Owing to the stochastic nature of MOT, each algorithm was run 20 times. The results in Table 3 are given in terms of average value, and standard deviation. Both of these, in conjunction with the convergence rate, are critical parameters in the analysis of MOT.

**Table 3:** Comparison of PSO, ABC and WOA for three benchmark functions at 5, 50 and 100D.

Function	Dimension		PSO	ABC	WOA
1	5	Mean	364	0.08	32.42
		Std	499	0.07	98.69
		Rank	3	1	2
2		Mean	0.54	0.17	0.14
		Std	0.23	0.04	0.14
		Rank	3	2	1
3		Mean	28.23	4.22	1.44
		Std	12.21	1.08	6.47
		Rank	3	2	1
1	50	Mean	7.76E+05	30.50E+05	3.34E+05
		Std	6.03E+05	10.36E+05	6.68E+05
		Rank	2	3	1
2		Mean	1.15	6.12	0.05
		Std	0.07	0.83	0.16
		Rank	2	3	1
3		Mean	604.39	650.64	181.82
		Std	73.16	43.91	154.84
		Rank	2	3	1
1	100	Mean	2.59E+06	27.12E+06	5.58E+05
		Std	1.97E+06	3.37E+06	1.28E+06
		Rank	2	3	1
2		Mean	1.33	25.46	5.55E-18
		Std	0.18	1.69	2.48E-17
		Rank	2	3	1
3		Mean	1390.33	1644.30	645.32
		Std	120.28	36.98	174.59
		Rank	3	2	1

Considering the results of the 5 dimension set, it is observed that for F1, the ABC yields the best average value. This is succeeded by the WOA. For F2 and F3, the WOA produces the best result, with PSO producing the poorest average value. However, for these results, the ABC showed a superior standard deviation to the WOA. This indicates an inferiority of WOA to ABC in terms of performance stability. For the results of the 50 dimension set, the WOA generated the best average value with the ABC having the poorest response. For F1 and F2, PSO showed greater stability to WOA, while for F3 the WOA produced the poorest stability performance. As with the 50 dimension set, the WOA yielded a superior average value for all three functions, with the ABC producing the poorest result. Considering stability, WOA is superior for F1 and F2, but is inferior to both PSO and ABC for F3.

**Figure 24:**
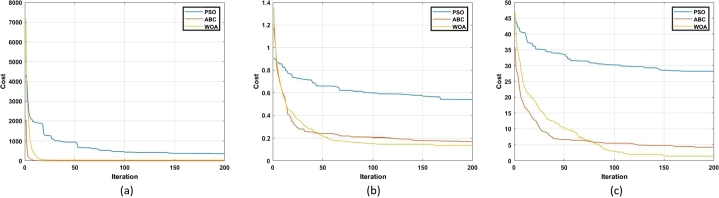
Convergence curves of algorithms at 5D for (a) F1, (b) F2 and (c) F3.

**Figure 25:**
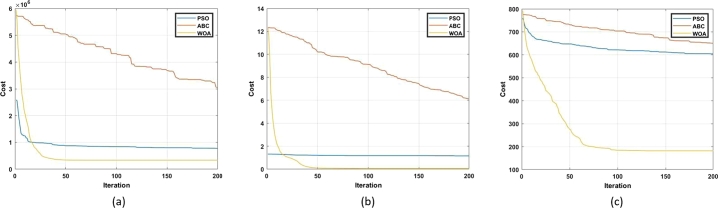
Convergence curves of algorithms at 50D for (a) F1, (b) F2 and (c) F3.

**Figure 26:**
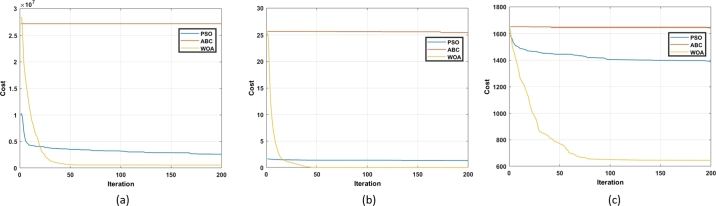
Convergence curves of algorithms at 100D for (a) F1, (b) F2 and (c) F3.

Figure 24, Figure 25, Figure 26 depict the convergence curves of each algorithm, for each test function and at each of the dimension magnitudes utilized. Considering Fig. 24, for F1, ABC yielded the best convergence rate, with PSO yielding the worst in this aspect. For F2, once again PSO generated the poorest response. ABC is superior to WOA until about 45 iterations, after which WOA outperforms the former. A similar result is seen for F3, with the different being that the superiority of WOA presenting at 75 iterations.

Considering Fig. 25, for F1, ABC generated the poorest response. PSO proved to be superior to WOA until about 20 iterations, after which WOA exhibits dominancy. A near identical trend is observed for F2. This is not the case for F3, where WOA is superior, and ABC once again yielding the poorest result. Considering Fig. 26, for F1, ABC generated the poorest response. PSO exhibited dominancy to WOA until about 25 iterations, after which WOA proved to be superior. A similar trend is observed for F2, this time the WOA obtaining dominancy at a slightly smaller iteration count. For F3, WOA proved to be superior, and ABC once again yielding the poorest result.

## 7 Conclusion

This paper provided a review on swarm-based Metaheuristic Optimization Techniques in terms of algorithm structure, merits, demerits, and application to the control of the DFIG. While there exist numerous swarm-based Metaheuristic Optimization Techniques, only fourteen techniques were covered in this paper. The swarm-based techniques which featured in this paper were PSO, BFO algorithm, ABC optimization, GWO, BA, SSA, CuSA, FA, MFO, SFO, ALO, SFLA, CSA and WOA. The theory behind these algorithms, as well as their mathematical models, were provided. It was seen that while all these algorithms differ in terms of structure and method of optimization, they share some commonalities. The biggest commonality is the stochastic nature of the algorithms. All these metaheuristic optimization techniques rely on the use of randomized numbers, usually between 0 and 1.

When considering applications of swarm-based metaheuristic optimization techniques, there exist various examples in engineering. While the techniques provide strong performances in general, many of the conventional algorithm suffer the demerits of a poor rate of convergence and being easily entrapped in the local minima. However, the good news is that these problems have been ameliorated in many of the algorithms. The demerits of the SFO algorithm and BFO algorithm have not yet been discovered. Based on the observable demerits of the other algorithms, this can be successfully investigated. However, when it considering the application of these techniques to energy generation systems, not many examples exist. Considering application to the DFIG, only particle swarm optimization has been researched and applied somewhat thoroughly. Some algorithms like GWO, ABC optimization, BFO algorithm, CuSA, FA, ALO, MFO, CSA and BA have only been applied once or twice. Other algorithms like SFLA, SSA and SFO are yet to be applied to the DFIG. When applied to the DFIG, swarm-based MOT have produced good results. However, due to the lack of application and rigorous testing of these techniques, extensive testing is required to validate their effectiveness. Hence it would be beneficial to research and apply the algorithms, especially those which are yet to be done so. Upon completion of this article, the authors propose the following future scope of work to be completed:

• An investigation into the demerits of BFO and SFO and measures to overcome possible demerits.
• The application of the SFLA, SSA and SFO to the control of the DFIG.
• The application of the modified swarm-based MOT, which are discussed in this paper, to the control of the DFIG.
• An investigation into other swarm-based MOT. This is in terms of structure, mathematical modelling, shortcomings, advancements, and application to the control of the DFIG.
• An investigation into physics-based algorithms, evolution-based algorithms, and human related algorithm. This is in terms of structure, mathematical modelling, shortcomings, advancements, and application to the control of the DFIG.
• The combining of MOT and thereby creating hybrid algorithms to be applied to the DFIG. This would be for the intention of combining the merits of two algorithms and thereby eliminating the demerits of such algorithms.

## Declarations

### Author contribution statement

All authors listed have significantly contributed to the development and the writing of this article.

### Funding statement

This research did not receive any specific grant from funding agencies in the public, commercial, or not-for-profit sectors.

### Data availability statement

No data was used for the research described in the article.

### Declaration of interests statement

The authors declare no conflict of interest.

### Additional information

No additional information is available for this paper.

## Figures and Tables

**Table:** 

Function number	Function name	Description	Range	Optimum
F1	DixonPrice	$\begin{array}{c} {\left(x_{1}-1\right)}^{2}\\ +\sum_{i=2}^{d}i{\left(2x_{i}^{2}-x_{i-1}\right)}^{2} \end{array}$	[−10,10]	0
F2	Griewank	$\begin{array}{c} 1+\frac{1}{4000}\sum_{i=1}^{d}x_{i}^{2}\\ -\sum_{i=1}^{d}\cos(\frac{x_{i}}{\sqrt{i}}) \end{array}$	[−60,60]	0
F3	Rastrigin	$\begin{array}{c} 10d\\ +\sum_{i=1}^{d}[x_{i}^{2}-10\cos(2\pi x_{i})] \end{array}$	[−5.12,5.12]	0
